# A generalized mathematical framework for the calcium control hypothesis describes weight-dependent synaptic plasticity

**DOI:** 10.1007/s10827-025-00894-6

**Published:** 2025-03-18

**Authors:** Toviah Moldwin, Li Shay Azran, Idan Segev

**Affiliations:** 1 Edmond and Lily Safra Center for Brain Sciences, The Hebrew University of Jerusalem, Jerusalem, Israel; 2 Department of Brain Sciences, Weizmann Institute of Science, Rehovot, Israel; 3 Department of Neurobiology, The Hebrew University of Jerusalem, Jerusalem, Israel

**Keywords:** Synaptic weights, Synaptic plasticity, Calcium, Calcium-based plasticity, Neural plasticity, Protein synthesis, Place fields, Place cells, BTSP, STDP

## Abstract

The brain modifies synaptic strengths to store new information via long-term potentiation (LTP) and long-term depression (LTD). Evidence has mounted that long-term synaptic plasticity is controlled via concentrations of calcium ([Ca^2+^]) in postsynaptic dendritic spines. Several mathematical models describe this phenomenon, including those of Shouval, Bear, and Cooper (SBC) (Shouval et al., 2002, 2010) and Graupner and Brunel (GB) (Graupner & Brunel, 2012). Here we suggest a generalized version of the SBC and GB models, the fixed point – learning rate (FPLR) framework, where the synaptic [Ca^2+^] specifies a fixed point toward which the synaptic weight approaches asymptotically at a [Ca^2+^]-dependent rate. The FPLR framework offers a straightforward phenomenological interpretation of calcium-based plasticity: *the calcium concentration tells the synaptic weight where it is going and how quickly it goes there.* The FPLR framework can flexibly incorporate various experimental findings, including the existence of multiple regions of [Ca^2+^] where no plasticity occurs, or plasticity observed experimentally in cerebellar Purkinje cells, where the directionality of calcium-based synaptic changes is reversed relative to cortical and hippocampal neurons. We also suggest a modeling approach that captures the dependency of late-phase plasticity stabilization on protein synthesis. We demonstrate that due to the asymptotic nature of synaptic changes in the FPLR rule, the plastic changes induced by frequency- and spike-timing-dependent plasticity protocols are weight-dependent. Finally, we show how the FPLR framework can explain the weight-dependence observed in behavioral time scale plasticity (BTSP).

**Supplementary Information:**

The online version contains supplementary material available at 10.1007/s10827-025-00894-6.

## Introduction

Since the work of Donald Hebb (Hebb, 1949), it has been believed that the brain learns via modifying the strengths of synaptic connections between neurons. Decades of experimental research have shown that synaptic strengths can be increased via a process known as long-term potentiation (LTP) and decreased via another process known as long term depression (LTD). Experimentally, there are a variety of protocols that can induce either potentiation or depression, usually involving stimulating the presynaptic inputs (e.g., via electrical or optical stimulation of presynaptic axons, or glutamate uncaging at the synapse), the postsynaptic soma (e.g., by electrically inducing subthreshold depolarization or spiking), or both, for varying frequencies and durations (Shouval et al., 2010).

Over the past several decades, plasticity researchers have been drawn to the possibility that there may be a fundamental molecular process underlying long-term plasticity. Lisman (Lisman, 1989) proposed a calcium-based theory of plasticity, an idea which has subsequently been extensively experimentally validated (Cho et al., 2001; Cummings et al., 1996; Lisman, 1989; Shouval et al., 2002; Yang et al., 1999). In this framework, different presynaptic and postsynaptic stimulation protocols differentially yield potentiation or depression due to the amount of calcium which flows into the postsynaptic area as a consequence of the stimulation. Specifically, if the calcium concentration ([Ca^2+^]) is low, no change occurs to the synaptic strength. If the [Ca^2+^] rises above a critical threshold for depression (${\theta }_{D}),$ long-term depression (LTD) occurs and the synaptic strength is decreased. If the [Ca^2+^] rises above the critical threshold for potentiation $({\theta }_{P}$), long-term potentiation (LTP) occurs and the synaptic strength is increased. There is evidence that calcium promotes LTP via pathways involving protein kinases such as calmodulin kinase (CaMKII) (Lisman, 1989; Malenka et al., 1989; Neveu & Zucker, 1996; R et al., 1989), while promoting LTD via phosphatases such as calcineurin (Lisman, 1989; Mulkey et al., 1994; Mulkey et al., 1993).

The success of the calcium theory of plasticity, also known as the *calcium control hypothesis*, has attracted theoretical neuroscientists and modelers to attempt to capture the dynamics of calcium-based plasticity in equations and computer models. Two models commonly in use are the models of Shouval, Bear, and Cooper (SBC)(Shouval et al., 2002, 2010) and the model Graupner and Brunel (GB)(Graupner & Brunel, 2012). There are several differences between these models, and we will explore them later. Fundamentally, though, both models attempt to capture 3 essential properties:

1. Synaptic weights decrease when the synaptic [Ca^2+^] is in the depressive calcium region (${\theta }_{D} \le [{Ca}^{2+}] \le {\theta }_{P})$ and potentiate when the [Ca^2+^] is in the potentiative calcium range ($\left[{Ca}^{2+}\right]> {\theta }_{P}$).
2. Synaptic weights do not potentiate or decrease linearly ad infinitum in the presence of potentiating or depressing levels of calcium, but rather they increase or decrease asymptotically toward some maximum or minimum value. This accounts for the fact that in biology synaptic weights cannot be arbitrarily large or arbitrarily small (excitatory synapses can’t have negative weights) and the fact that synaptic strengths are observed to exhibit saturating behavior when undergoing a plasticity protocol (O’Connor et al., 2005b).
3. In the pre-depressive region of $([Ca^{2+}] < \theta_D)$, neither potentiation nor depression occur, but synaptic strengths may still “drift” toward some pre-determined value(s), called stable states or fixed points, over a long time scale (i.e. hours or days). This feature is motivated by evidence that synapses may have a discrete number of stable states and that synaptic strengths have been experimentally observed to slowly drift toward stable states after inducing potentiation or depression strengths. This slow drift after plasticity induction is also known as the "late phase" of plasticity (Frey & Morris, 1997; Kauderer & Kandel, 2000; Manahan-Vaughan et al., 2000; Redondo et al., 2010; Sajikumar et al., 2005). (The major practical difference between the SBC model and the GB model pertains to this late phase. In the SBC model, weights drift towards a single baseline during the late phase whereas in the GB model, synapses that are above a certain weight are stabilized toward an UP state while synapses that are below that weight are stabilized toward a DOWN state.)

In this work, we will review and compare the SBC and GB models and make several modifications to both models to make them more analytically straightforward, thus allowing both experimentalists and theoreticians to use them with greater ease. Our modifications are based on the principle that a calcium-based plasticity model should allow for explicit and independent specification of critical parameters that capture the essential dynamics of the plasticity. Specifically, it should be possible to specify the fixed points of the asymptotic dynamics of the synaptic weights in each region of the [Ca^2+^] space (and in each region of the weight space in the GB rule), and it should also be possible to independently specify the rates (time constants) at which potentiation, depression, and drift take place without constraining the fixed points. We therefore call our model the fixed point – learning rate (FPLR) framework, as its dynamics can be fully characterized by specifying the fixed points and learning rates (time constants).

The FPLR framework is a phenomenological—as opposed to mechanistic—model of plasticity. The underlying assumption is that different levels of calcium specify different processes in the cell that have different teleological endpoints (i.e., the fixed points of the weights) and that occur at different rates. In other words, *the calcium concentration tells the synaptic weight where it is going and how quickly it goes there*.

**Fig. 1:**
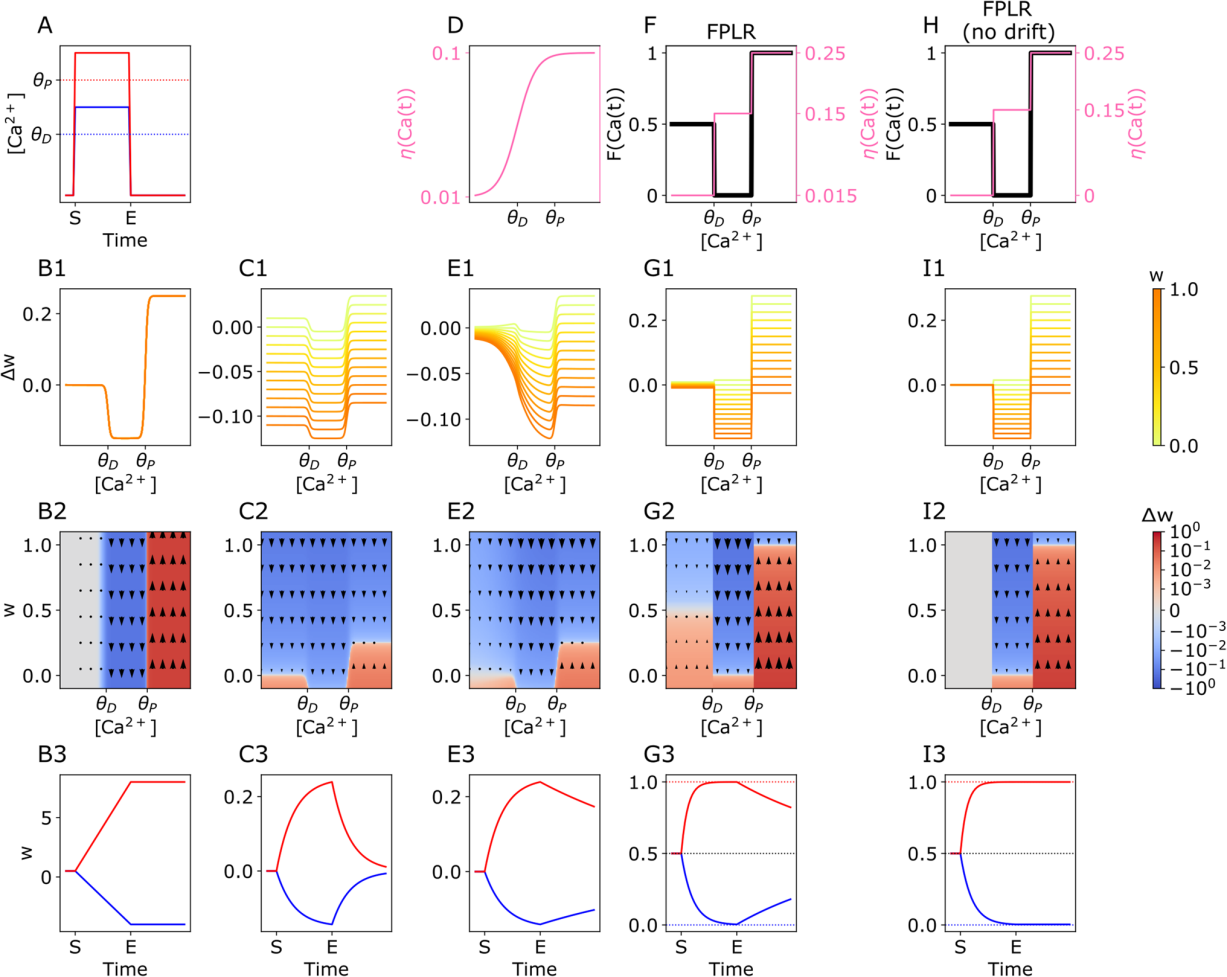
SBC and FPLR rules for Calcium-based plasticity. **(A)** Basic stimulation to test the plastic effect of different levels of $\left[{Ca}^{2+}\right]$. At time S (Start), $\left[{Ca}^{2+}\right]$ is either raised to a depressive level (${\theta }_{D} \le \left[{Ca}^{2+}\right]\le {\theta }_{P}$, blue line) or to a potentiative level ($\left[{Ca}^{2+}\right]> {\theta }_{P}$, red line) for several minutes until time E (end) and is then reduced to and held at 0 for several hours to observe calcium-independent drift effects. **(B1)** Linear SBC two-threshold rule for $\left[{Ca}^{2+}\right]$-dependent weight changes. **(B2)** Same as (B1) but with $\Delta w$ presented as a heatmap of both the present value of $w$ and the $\left[{Ca}^{2+}\right]$. In the linear SBC rule, there is no dependence on the present value of $w$ and thus no variation along the vertical axis. Colors are log-normalized for visualization purposes. Red indicates potentiation, blue indicates depression, white indicates no change. **(B3)** Resultant weights over time when applying the linear SBC rule to the stimulation protocol from (A). Red and blue lines correspond to the red and blue lines (potentiative and depressive protocols, respectively) from (A). In the linear SBC rule, synapses potentiate or depress linearly in the presence of calcium and remain stable in the absence of calcium. **(C1)** SBC rule with weight decay. Here $\Delta w$ depends on both the present value of $w$ and the ${[Ca}^{2+}]$. Larger weights (darker lines) depress faster and potentiate more slowly than lower weights (lighter lines, some weight values are negative for illustrative purposes). **(C2)** Same as (C1), presented as a heatmap. White line indicates fixed points. **(C3)** Weights over time in the SBC rule with weight decay. Dotted horizontal lines indicate fixed points. **(D)** Calcium-dependent learning rate in the original SBC rule. The rate of weight change increases sigmoidally with the ${[Ca}^{2+}]$, allowing for a slower drift to baseline for pre-depressive levels of ${[Ca}^{2+}]$. **(E1-E3)** Dynamics of SBC rule with weight decay and $\left[{Ca}^{2+}\right]$-dependent learning rate. **(F)** Fixed points ($F\left(Ca\left(t\right)\right)$, black line, left y-axis) and learning rates ($\eta \left(Ca\left(t\right)\right)$, pink line, right y-axis) for each region of ${[Ca}^{2+}]$ as step functions in the FPLR rule. **(G1-G3)** Dynamics of the FPLR rule. **(H)** Fixed points and learning rates for the FPLR without drift. $\eta \left(Ca\left(t\right)\right)$ is set to 0 in the pre-depressive region of $\left[{Ca}^{2+}\right].$**(I1-I3)** Dynamics of the FPLR rule with no drift

The dynamics of calcium-based plasticity can be captured by performing experiments where the synaptic [Ca^2+^] is held at a fixed level for some duration of time (on the order of minutes) until the synaptic weight reaches an asymptote, and then decreased to 0 for a longer duration of time (on the order of hours) this protocol reveals the fixed points and the rate at which they are reached in both the early and late phases of plasticity (Fig. 1A).

With this canonical experiment in mind, we will review the original SBC and GB rules and replace them, respectively, with “one-dimensional” and “two-dimensional” rules using the FPLR framework. While doing so, we will point out some subtle properties of the original rules and their FPLR counterparts. We will also show how the FPLR rules can be used to describe a wide range of experimental findings in plasticity research.

## Results

### Linear SBC rule

In general, we would like to find a rule that describes the change in a synaptic weight over time as a function of the calcium concentration, i.e. some differential equation of the form $\frac{dw}{dt}=f\left(Ca\left(t\right)\right)$, where $w$ is the synaptic weight, $Ca\left(t\right)$ is the calcium at that synapse at time $t$, and $f$ is some function. It is also possible to use a discrete-time formulation appropriate for numerical computer simulation using a first-order Taylor approximation, where the value of the weight at each time step, $w\left(t\right)$, is equal to the weight at the previous time-step plus a modification that depends both on the present weight and the weight at the current time step, i.e. $w\left(t\right)=w\left(t-1\right)+\Delta w$, where $\Delta w= f\left(Ca\left(t\right), w\left(t\right)\right)$. (In the equations below, for parsimony, we will generally write $w$ instead of $w\left(t\right)$, but the synaptic weight is always a function of time).

We first consider the calcium-based plasticity rules of (Shouval et al., 2002), starting with the simplest formulation. In this rule, synaptic strength is modified in a straightforward manner according to the depression and potentiation thresholds: at any time step $t$, if the calcium concentration [Ca^2+^] is in the pre-depressive range ($\left[{Ca}^{2+}\right]< {\theta }_{D}$) the synaptic weight $w$ remains unchanged. If the calcium concentration is in the depressive range (${\theta }_{D} \le \left[{Ca}^{2+}\right]\le {\theta }_{P}$), $w$ is decreased, and if the [Ca^2+^] is in the potentiating range ($\left[{Ca}^{2+}\right]> {\theta }_{P}$), $w$ is increased. Formally, the change in the synaptic weight $\Delta w$ is given by:

2.1.1 $$\Delta w=\eta \Omega \left(Ca\left(t\right)\right)$$

where $\eta$ is the learning rate and $\Omega$ is the two-threshold calcium based plasticity function described above, which can be expressed most simply as a step function:

2.1.2 $$\Omega\left(Ca\left(t\right)\right)=\left\{\begin{array}{l}\begin{array}{lc}0,&\;\;Ca(t)<\theta_D\end{array}\\\begin{array}{lc}k_D,&\theta_D\leq Ca(t)\leq\theta_P\end{array}\\\begin{array}{lc}k_P,&Ca(t)>\theta_P\end{array}\end{array}\right.$$

where $${k}_{D}$$ and $${k}_{P}$$ are the signed rates of depression and potentiation, respectively ($${k}_{D}<0, {k}_{P}>0)$$, and $${\theta }_{D}$$ and $${\theta }_{P}$$ represent the thresholds for depression and potentiation. If smooth transitions between regions are desired, this can also be expressed with a soft threshold using the sum of sigmoids (slightly modified from Shouval et al., 2002):

2.1.3 $$\Omega \left(Ca\left(t\right)\right)=\frac{{k}_{D}}{1+{e}^{-{b}_{D}\left(Ca\left(t\right)-{\theta }_{D}\right)}}+\frac{{k}_{P}{-k}_{D}}{1+{e}^{-{b}_{P}\left(Ca\left(t\right)-{\theta }_{P}\right)}}$$

where ${b}_{\text{D}}$ and ${b}_{\text{P}}$ control the sharpness of the transitions between regions in the $\Omega$ function.

In the linear SBC rule, synaptic weights increase linearly at a rate of ${k}_{P}$ in the potentiative region of $\left[{Ca}^{2+}\right]$, decrease linearly at rate of ${k}_{D}$ in the depressive region of $\left[{Ca}^{2+}\right]$ and remain stable in the pre-depressive region of $\left[{Ca}^{2+}\right]$. The change in synaptic weight depends only on the calcium concentration, not on the present value of the synaptic weight (Fig. 1B1-1B2). When applying our canonical calcium step stimuli or a potentiative or depressive level of calcium, we observe that the synaptic weights linearly increase or decrease while the calcium stimulus is active, and then the weights cease to change when the $\left[{Ca}^{2+}\right]$ is dropped to 0 (Fig. 1B3).

(We note that it may be more biologically plausible to implement the plasticity as a delayed function of the calcium signal, in which case one may substitute $Ca\left(t-D\right)$ in place of $Ca\left(t\right)$, where $D$ indicates the duration of the temporal delay between the calcium signal and the plastic effect. For simplicity, however, we will assume that there is no such delay, i.e. $D=0$.)

### SBC rule with weight decay

To prevent synapses becoming arbitrarily large or small, (Shouval et al., 2002) added a weight decay term to the original plasticity rule.

2.2.1 $$\Delta w=\eta \left(\Omega \left(Ca\left(t\right)\right)- \lambda w\right)$$

where $\lambda$ is the rate of decay. Importantly, the change in the weight $\Delta w$ now depends on both the [Ca^2+^] and the present value of the weight $w$. It can be instructive to visualize $\Delta w$ as a function of both $w$ and [Ca^2+^]. We show how the SBC rule with weight decay differs from the rule without it. When weight decay is added, the magnitude of the weight change depends both on the calcium level and the current weight (Fig. 1C1-1C2).

The difference in dynamical behavior between the SBC rule with weight decay (Eq. 2.2.1) and the linear version (Eq. 2.1.1.) become clear when applying the calcium step experiment described above. In the linear SBC rule without weight decay, the weight linearly increases (or decreases) for as long as the calcium pulse is present, then immediately stops changing (i.e., remains stable) when the calcium is turned off. By contrast, when weight decay is used, in the pre-depressive range of [Ca^2+^], $w$ decreases (or increases, if it is negative) asymptotically to the fixed point of 0 at a rate of $\eta\uplambda$. In the depressive [Ca^2+^] range, $w$ decreases asymptotically to the fixed point of $\frac{{k}_{D}}{\uplambda }$ (or increases if the weight is below that point). In the potentiating range of [Ca^2+^], $w$ increases asymptotically to the fixed point of $\frac{{k}_{P}}{\uplambda }$ (or decreases if the weight is above the fixed point) (Fig. 1C3). Note that in this framework, there is no way to independently change the asymptotic behavior without changing either the rates of potentiation and depression (${k}_{D}$ and ${k}_{P}$) or the decay rate $\uplambda$.

### *SBC rule with weight decay and Ca*^*2*+^*-dependent learning rate*

Weight decay that drifts quickly toward 0 in the absence of plasticity-inducing calcium may be an undesirable feature of a plasticity rule if we wish to model synapses that are potentiated or depressed for a long duration. (Shouval et al., 2002) therefore introduced a sigmoidal [Ca^2+^]-dependent learning rate, $\eta \left(Ca\left(t\right)\right)$, to mitigate the effect of the weight decay in the absence of calcium. The SBC rule with the calcium-dependent learning rate is thus defined as:

2.3.1 $$\Delta w=\eta \left(Ca\left(t\right)\right)*(\Omega \left(Ca\left(t\right)\right)- \lambda w)$$

The basic idea is that instead of having a constant learning rate, the learning rate (including the rate of the weight decay) increases in a sigmoidal fashion with the amount of calcium (Fig. 1D). Thus, at pre-depressive levels of [Ca^2+^] the weight will decay slowly, allowing for greater stability over long time horizons. (Fig. 1E1-1E3).

### Fixed point – learning rate version of the SBC rule

In the FPLR framework, we propose a modified version of the SBC rule which allows the modeler to specify the fixed points and learning rates explicitly in all three regions of [Ca^2+^]:

2.4.1 $$\Delta w=\eta \left(Ca\left(t\right)\right)*\left(F\left(Ca\left(t\right)\right)-w\right)$$

Here, instead of $\Omega \left(Ca\left(t\right)\right)$, we use $F\left(Ca\left(t\right)\right)$, a step function which describes the *fixed points* of the weights as a function of the [Ca^2+^]. (We note that if $\lambda$ is fixed to 1 in the original SBC rule [Eq. 2.3.1)], $\Omega \left(Ca\left(t\right)\right)$ also specifies the fixed points of the weights, however $F\left(Ca\left(t\right)\right)$ is defined this way explicitly.) Similar to the SBC rule, $\eta \left(Ca\left(t\right)\right)$ here is also a 3-valued step function which determines the rate at which the weight asymptotically approaches the fixed point for each calcium level. (We require $\eta \left(Ca\right)\le 1$ for all values of $Ca$ to prevent oscillations; $\eta \left(Ca\left(t\right)\right)=1$ is a special case in which the synapse immediately jumps to the fixed point specified by $F\left(Ca\left(t\right)\right)$, which can be useful for modeling discrete-state synapses.)

In this framework, the learning rate $\eta \left(Ca\left(t\right)\right)$ defines the fraction of the difference between the current weight and the fixed point $F\left(Ca\left(t\right)\right)$ which is traversed at each time step. We call this the “one-dimensional” version of the FPLR rule, because both the fixed points and the learning rates depend only the calcium concentration at that time step.

For example, we might have:

2.4.2 $$F\left(Ca(t)\right)=\left\{\begin{array}{ll}0.5,&Ca(t)<\theta_D\\0,&\theta_D\leq Ca(t)<\theta_P\\1,&Ca(t)\geq\theta_P\end{array}\right.$$

And

2.4.3 $$\eta\left(Ca(t)\right)=\left\{\begin{array}{ll}0.015,&Ca(t)<\theta_D\\0.15,&\theta_D\leq Ca(t)<\theta_P\\0.25,&Ca(t)\geq\theta_P\end{array}\right.$$

This means that synapses with pre-depressive calcium concentrations ($Ca\left(t\right)< {\theta }_{D}$) eventually drift toward a “neutral” state of 0.5 at a rate of 0.015, synapses with a depressive calcium concentration (${\theta }_{D}\le Ca\left(t\right)<{\theta }_{P}$) will depress towards 0 at a rate of 0.15, and synapses with a potentiative [Ca^2+^] ($Ca\left(t\right)\ge {\theta }_{P}$) will be potentiated towards 1 at a rate of 0.25 (Fig. 1F, 1G). (See (Enoki et al., 2009) for experimental evidence that synapses at baseline can be either potentiated or depressed. Note that the fixed points and rates used here and in subsequent sections are specified in arbitrary units and meant to illustrate qualitative dynamics of the synapse only; see Fig. 5 and **Methods** for biologically plausible parameters).

The FPLR rule is structurally similar to the SBC rule with weight decay and calcium-dependent learning rate (Eq. 2.3.1) (Fig. 1D-E). The advantage of the FPLR formulation, however, is the explicit interpretation of each piece of the equation. In the FPLR formulation there is no ‘weight decay’, there are rather only fixed points $F\left(Ca\left(t\right)\right)$, and learning rates, $\eta \left(Ca\left(t\right)\right)$, which are independently specified.

We can also turn off the drift in the pre-depressive region entirely by setting $\eta \left(Ca\left(t\right)<{\theta }_{D}\right)=0$, thus allowing for synapses that are stable at every weight unless modified by depressive or potentiative calcium concentrations (Fig. 1H,1I). This version of the rule can be useful for contexts in which the modeler is primarily interested in understanding the early phase of plasticity, or for theoretical work exploring the consequences of synaptic weights that potentiate and depress asymptotically. (Turning off the pre-depressive drift can also potentially affect the behavior of the synaptic weights in the early phase of plasticity in plasticity protocols where synaptic stimulation is relatively slow, e.g. on the order of 1 Hz. However, because the time scale of the drift is 2–3 orders of magnitude slower than the time scale of the early phase plasticity, these effects are likely to be negligible when modeling plasticity during the early phase of plasticity.)

We note that if we use a differential equation interpretation of Eq. (2.4.1), i.e. we consider weight changes over time to be continuous such that $\frac{dw}{dt}=\Delta w$, it is possible to find a closed-form solution to the synaptic weight for a constant pulse of calcium of magnitude $C$ that starts at time ${t}_{S}$ and ends at time ${t}_{E}$:

2.4.4 $$w\left({t}_{E}\right)=F(C)+\left(w\left({t}_{S}\right)-F\left(C\right)\right){e}^{-\eta \left({t}_{E}-{t}_{S}\right)}$$

Equation (2.4.4) describes an exponential growth or decay process (depending on whether the target fixed point $F\left(C\right)$ is above or below the initial weight $w\left({t}_{S}\right)$) that asymptotically drives the synaptic weight toward the fixed point $F\left(C\right)$ with rate $\eta$. This behavior is apparent when we apply our calcium step stimuli. This formulation is also computationally useful; if we are interested in merely finding the final value of a synaptic weight following some extensive plasticity protocol and not calculating the weight change at each time point, it is only necessary to apply Eq. (2.4.4) once for each time the [Ca^2+^] crosses one of the plasticity thresholds to calculate the final synaptic weight value.

### Versatility of the FPLR framework

**Fig. 2:**
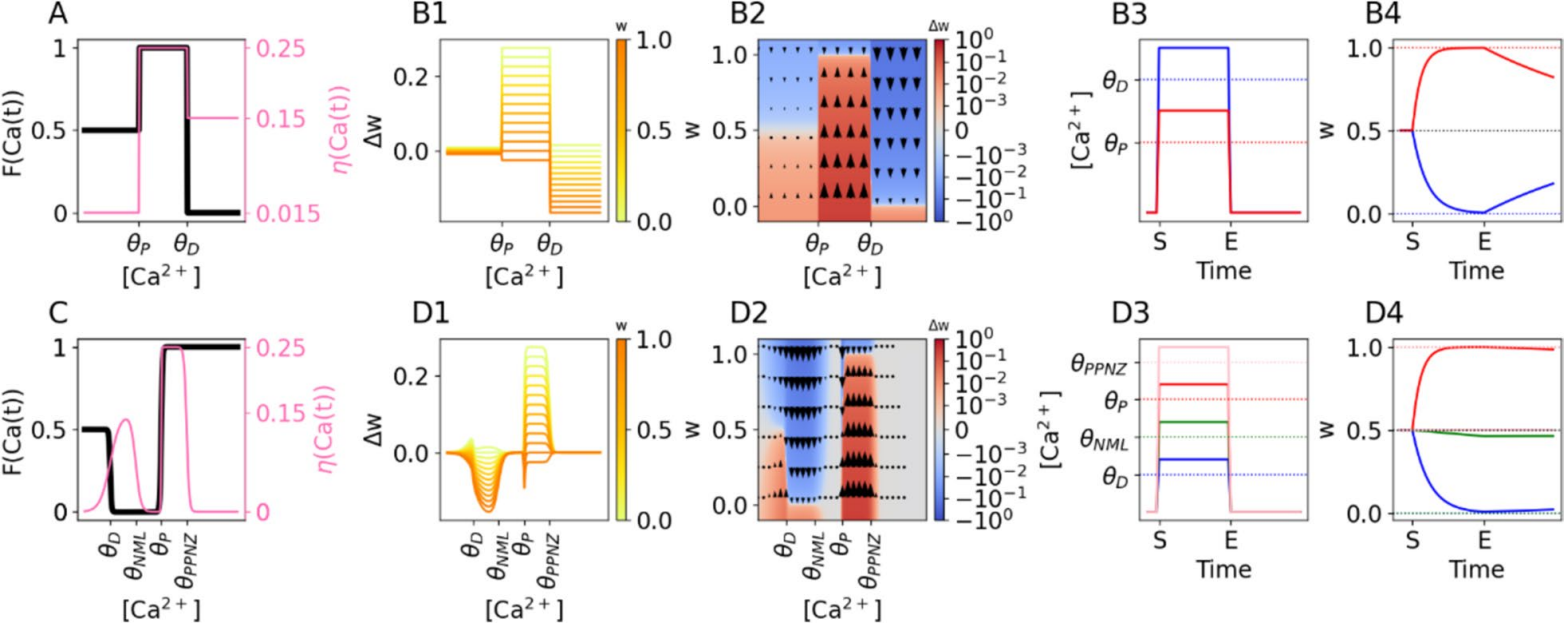
Incorporating multiple thresholds with the FPLR rule. **(A)** Possible fixed points and learning rates for Purkinje cells, where the potentiation threshold is lower than the depression threshold (${\theta }_{P}$< ${\theta }_{D})$.(**B**1) $\Delta w$ in modified SBC rule for Purkinje cells using the fixed points and learning rates from (A). **(B2)** Phase plane heatmap for Purkinje rule **(B3)** Stimulation protocol for Purkinje cells. Here, the $\left[{Ca}^{2+}\right]$ used for potentiation (red line) is less than the $\left[{Ca}^{2+}\right]$ used to induce depression. **(B4)** Weights over time in Purkinje cells for the stimulation protocols in (B3). Dotted lines represent fixed points. **(C)** Fixed points and learning rates, defined using a soft-threshold step function, with two additional regions of $\left[{Ca}^{2+}\right]$ where $\eta =0$ and therefore no plasticity is induced. ${\theta }_{NML}$: no man’s land, ${\theta }_{PPNZ}$: post-potentiative neutral zone. **(D1)** $\Delta w$ in FPLR rule using the fixed points and learning rates from (C). Note the U-shaped dependence of depression magnitude on the $\left[{Ca}^{2+}\right]$ resulting in a no man’s zone between the depressive and potentiative regions of $\left[{Ca}^{2+}\right]$. Irregularities near the boundaries of $\left[{Ca}^{2+}\right]$ regions are due to the soft transitions in the fixed point and plasticity rate functions. **(D2)** Phase plane heatmap for rule shown in (C). **(D3)** Stimulation protocol for each region of $\left[{Ca}^{2+}\right]$ shown in (F). **(D4)** Weights over time for the stimulation protocols in (D3). Note that when the $\left[{Ca}^{2+}\right]$ is in the no man’s land (green line) depression occurs at a much slower rate, and in the post-potentiative neutral zone (black line) nearly no plasticity occurs

Until now, all of the above rules have assumed that there are only three regions of [Ca^2+^] relevant for plasticity and that the depression threshold is lower than the potentiation threshold, i.e. ${\theta }_{D}<{\theta }_{P}$. There are some experimental results that complicate this picture. For example, there is evidence that in Purkinje neurons, the depression threshold is higher than the potentation threshold, i.e. ${\theta }_{D}>{\theta }_{P}$ (Coesmans et al., 2004). This can also easily be implemented in the FPLR rule by changing the fixed points in each [Ca^2+^] region. (Fig. 2A, 2B).

Moreover, even within hippocampal and cortical cells, there may be additional regions of [Ca^2+^] where the plasticity dynamics change. Cho et al. (Cho et al., 2001) found that within the depressive region of [Ca^2+^], the magnitude of depression exhibits a U-shaped relationship with the calcium concentration, such that a “No man’s land” appears at the boundary between the depressive and potentiative region of [Ca^2+^], where the [Ca^2+^] is too large to induce depression but too small to induce potentiation (Cho et al., 2001; Lisman, 2001). It may make sense for a modeler to include this no-man's land more explicitly in the plasticity rule. There is also some evidence (see Fig. 3 in Tigaret et al., 2016) that there is some maximum level of [Ca^2+^] beyond which potentiation mechanisms are inactivated. It would be worthwhile to be able to incorporate this “post-potentiative neutral zone” as well. These “neutral zones” can easily be incorporated into the FPLR by adding additional thresholds into the step function from Eq. (2.4.1) and setting the learning rate to 0 in those regions (the fixed points in the no-plasticity regions can be chosen arbitrarily, as they are irrelevant if the learning rate in those regions is 0).

**Fig. 3:**
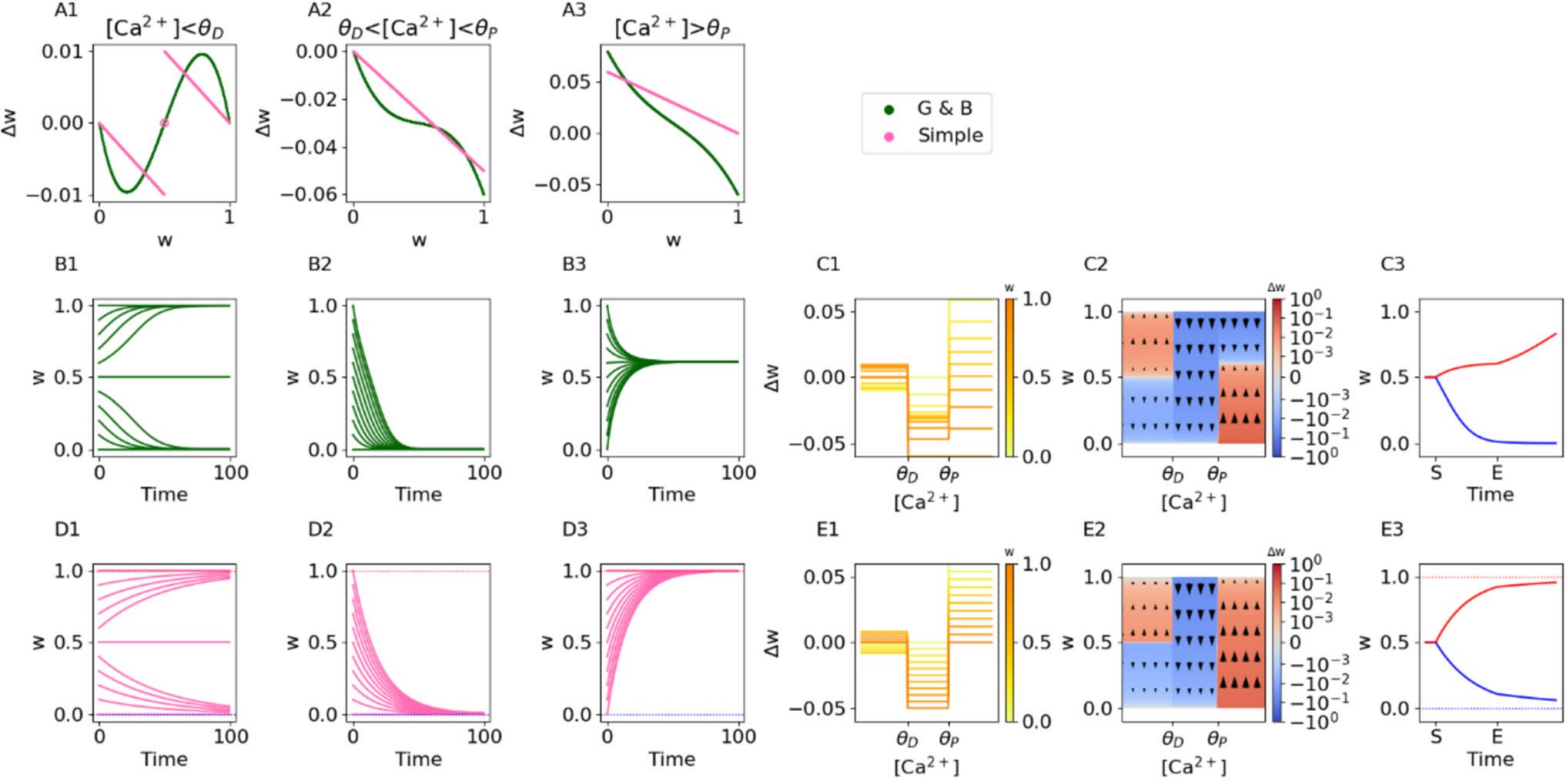
Graupner-Brunel rule and simplification. **(A1-A3)** $\Delta w$ as a function of $w$ for the original GB rule (green line) and for the simplified GB rule (pink line) in the pre-depressive, depressive, and potentiative regions of $\left[{Ca}^{2+}\right]$, respectively. Pink circle in A1 indicates unstable fixed point. **(B1-B3)** Weights over time in the original GB rule in each region of calcium (as in A1-A3) for synapses with different initial weights. **(C1-C3)** Weight changes, phase plane heatmap, and response to step stimuli (See Figure 1A) in the GB model. Note that weights drift in different directions after the end of the stimulus due to their final weights at time E. **(D1-D3)** As in (B1-B3) for the simplified version of the GB model. **(E1-E3)** As in (C1-C3) for the simplified version of the GB model

If desired, we can also explicitly model the U-shaped dependency between [Ca^2+^] and synaptic depression. Although we have used a hard threshold step function to implement the previous examples of the FPLR rule, in principle both $F\left(Ca\left(t\right)\right)$ and $\eta \left(Ca\left(t\right)\right)$ can be arbitrary functions as long as $\eta \left(Ca\left(t\right)\right)<1$ for all values of $Ca\left(t\right)$. To implement a U-shaped region, we can extend the soft threshold step function from Eq. (2.1.3) into a general form to include arbitrary regions of [Ca^2+^]:

2.5.1 $$\eta \left(Ca\left(t\right)\right)= \sum_{i=1}^{N}\frac{{\eta }_{i}-{\eta }_{i-1}}{1+{e}^{-{b}_{i}\left(Ca\left(t\right)-{\theta }_{i}\right)}}+{\eta }_{0}$$

where ${\theta }_{1}\dots {\theta }_{i}\dots {\theta }_{N}$ are the thresholds that differentiate between regions of [Ca2 +] ordered such that ${\theta }_{i}< {\theta }_{i+1}$, ${{b}_{1}\dots b}_{i}\dots {b}_{N}$ determine the steepness of transition between each [Ca2 +] region, ${{\eta }_{1}\dots \eta }_{i}\dots {\eta }_{N}$ are the learning rates in each [Ca2 +] region, and ${\eta }_{0}$ is the learning rate in the region between 0 and ${\theta }_{1}$. This representation approaches the equivalent step function as the values for ${b}_{i}$ become sufficiently large. (A similar equation can be used for $F\left(Ca\left(t\right)\right)$). Using Eq. (2.5.1), we can easily create the U-shaped relationship between [Ca^2+^] and synaptic depression (Fig. 2C, 2D).

### Graupner and Brunel model

One drawback of the SBC plasticity rule (Eq. 2.3.1) is that even in the final version with a calcium-dependent learning rate, synaptic weights eventually trend toward 0 in the presence of pre-depressive levels of calcium. There is some experimental evidence, however, that synapses are bistable, existing in a potentiated (UP) state with weight ${w}_{UP}$ or a depressed (DOWN) state with weight ${w}_{DOWN}$, and that synaptic strengths slowly trend toward one of those two states depending on the early synaptic strength after inducing a plasticity protocol (Bagal et al., 2005; O’Connor et al., 2005a; Petersen et al., 1998). (Graupner & Brunel, 2012) proposed a model that captured these dynamics. In their model, the synaptic efficacy, $\rho$ ($\rho$ is linearly mapped to $w$ according to the equation $w= {w}_{DOWN}+ \rho \left({w}_{UP}- {w}_{DOWN}\right)$) asymptotically decreases to the DOWN state (at $\rho =0$) in the presence of depressive [Ca^2+^] or increases asymptotically to the UP state (near $\rho =1$) in the presence of potentiating [Ca^2+^]. When the [Ca^2+^] is pre-depressive, $\rho$ either increases toward 1 or decreases toward 0 on a very slow time scale depending only on the present value of $\rho$. Specifically, if $\rho$ is larger than the value of an unstable fixed point ${\rho }_{\star }$ (set to 0.5), $\rho$ trends toward the UP state whereas if $\rho$ is smaller than this value, $\rho$ trends toward the DOWN state. Formally, we have:

2.6.1 $$\begin{array}{c}\tau \frac{d\rho }{dt} = -\rho \left(1-\rho \right)\left({\rho }_{\star }-\rho \right)+ {\gamma }_{P}\left(1-\rho \right)\Theta \left[Ca\left(t\right)-{\theta }_{P}\right]\\ - {\gamma }_{D}\rho\Theta [Ca\left(t\right)- {\theta }_{D}]+ Noise(t)\end{array}$$

where $\tau$ is the overall time constant of synaptic change and ${\gamma }_{P}$ and ${\gamma }_{D}$ are parameters that denote the rate of potentiation and depression, respectively.

For consistency with the SBC rule, without loss of generality, we can replace $\rho$ in Eq. (2.6.1) with $w$ (${w}_{\star }$ is the unstable drift fixed point of $w$), replace ${\gamma }_{P}$ and ${\gamma }_{D}$ with ${\eta }_{P}$ and ${\eta }_{D}$, respectively ($\tau$ = $\frac{1}{\eta }$, representing a global time constant for the rule), and we can restate Eq. (2.6.1) as a discretized formulation expressed as a step function (ignoring the noise term):

2.6.2 $$\tau \Delta w = \left\{\begin{array}{ll}-w(1-w)(w_* - w), & Ca(t) < \theta_D \\-w(1-w)(w_* - w) - \eta_D w, & \theta_D \leq Ca(t) < \theta_P \\ -w(1-w)(w_* - w) - \eta_D w + \eta_P(1-w), & Ca(t) \geq \theta_P\end{array}\right.$$

The first line in Eq. (2.6.2) expresses the calcium-independent dynamics of the slow drift to the UP or DOWN state in a hyperbolic fashion depending on the value of $w$ (Fig. 3A1, 3B1, green lines). The second line in Eq. (2.6.2) contains two terms; the first term is the calcium-independent drift term from before, and the second term is only active while the [Ca^2+^] is above the depression threshold and describes the asymptotic depressive dynamics. Because the hyperbolic drift term is always active, it contributes somewhat to the dynamics of the weight change in the depressive region, although if ${\eta }_{D}>1$ the depressive dynamics will dominate the drift dynamics (Fig. 3A2, 3B2, green lines). The third line in Eq. (2.6.2) contains three terms: the first two terms are the drift and depressive terms, as before, and the final term describes the asymptotic potentiative dynamics. As such, both the drift term and the depressive term contribute to the dynamics of plasticity in the potentiative region of [Ca^2+^], but if ${\eta }_{P}>{\eta }_{D}$, the potentiative effect can dominate, but only for relatively low weights. (Fig. 3A3, 3B3, green lines).

The overall profile of the GB rule, as well as its response to calcium step stimuli, appear in (Fig. 3C1-3C3). When a depressive calcium step stimulus is applied to the synapse, the synapse starts depressing toward the DOWN state (0), and when the stimulus turns off, the weight continues to drift downward toward 0, but at a slower pace. When a potentiative stimulus is applied, however, the synaptic weight asymptotes at a point substantially below the UP value (1), because the fixed point in the potentiative region of [Ca^2+^] is affected by the depressive process which is still active during potentiation, as described above. Nevertheless, once the calcium step is turned off, the potentiated synapse drifts toward the UP value. (Fig. 3C3). This behavior differs from that of the SBC model and one-dimensional FPLR rule, where the potentiated synapse would also eventually drift down toward 0 after the potentiative stimulus is turned off.

### Simplified Graupner and Brunel model

While the GB rule (Eq. 2.6.2) can describe a variety of experimental results, its dynamics can be complicated by the fact that multiple processes are active simultaneously – that is, the slow calcium-independent drift of the first term is always active, and the depressive process is always active when the potentiation process is active. From a modeling standpoint, this aspect of the GB rule may not be desirable, as the dynamics in each region of [Ca^2+^] do not exhibit simple asymptotic behavior, the fixed point for potentiation is not trivial to specify (note that $w=1$ is not a fixed point of $w$ if $a\left(t\right)>{\theta }_{P}$, see Fig. 3B3), and specifying ${\gamma }_{P}$ is insufficient to know the actual rate of potentiation because the depressive term ${\gamma }_{D}$ also affects the potentiation rate.

From a biological standpoint, it is also questionable whether depressive and potentiating processes are active simultaneously. While some studies have shown that depressive and potentiative mechanisms are operative at the same time and compete with each other (Burrell & Li, 2008; O’Connor et al., 2005b), another study (Cho et al., 2001) argues that once the [Ca^2+^] reaches the potentiation threshold, the depressive mechanisms are turned off. The slow bistable drift mechanisms in the first term of Eq. (2.6.2) may also not be perpetually active; the long-term stabilization mechanisms required for late LTP/LTD (L-LTP and L-LTD) have been shown to be protein synthesis dependent, and this protein synthesis may only occur after the induction of early-LTP/LTD (Barco et al., 2008; Frey & Morris, 1997; Redondo et al., 2010).

We therefore propose a simplified version of the Graupner-Brunel rule which only has a single term active for any given concentration of calcium and synaptic efficacy value, resulting in a rule which achieves a qualitatively similar result but with more straightforward dynamics. Here, the rate of change within each region of the $\left[{Ca}^{2+}\right]$ is independently specified by its own learning rate (${\eta }_{drift}, { \eta }_{D},{\eta }_{P}$).

2.7.1 $$\Delta w=\left\{\begin{array}{ll}-\eta_{drift}w,&Ca(t)<\theta_D\;\text{and}\;w<w^\ast\\\eta_{drift}(1-w),&Ca(t)<\theta_D\;\text{and}\;w>w^\ast\\0,&Ca(t)<\theta_D\;\text{and}\;w=w^\ast\\-\eta_Dw,&\theta_D\leq Ca(t)<\theta_P\\\eta_P(1-w),&Ca(t)\geq\theta_P\end{array}\right.$$

In this rule, in the pre-depressive region of $\left[{Ca}^{2+}\right]$, $w$ drifts asymptotically (exponentially, not sigmoidally, as in the original GB rule) toward 0 at a rate of ${\eta }_{drift}$ if $w$ is below ${w}^{*}$ (first line), or asymptotically toward 1 at a rate of ${\eta }_{drift}$ if $w$ is above ${w}^{*}$ (second line). If w is exactly equal to ${w}^{*}$, no weight change occurs (unstable fixed point) (Fig. 3A1, 3D1, pink lines). In the depressive region of calcium (fourth line), $w$ trends asymptotically toward the fixed point of 0 at a rate of ${\eta }_{D}$ (Fig. 3A2, 3D2, pink lines), and in the potentiative region of calcium (fifth line), $w$ trends asymptotically toward the fixed point of 1 (unlike the original GB rule) at a rate of ${\eta }_{P}$ Fig. 3A3, 3D3, pink lines). The profile of this plasticity rule and its response to calcium step stimuli are shown in Fig. E1-E3. Note that when the potentiative calcium step is applied, in contrast to the original GB rule, the synaptic weight is potentiated asymptotically toward 1, and it continues to drift toward 1 after the stimulus is turned off (Fig. 1E3).

Astute readers may notice that the simplified GB rule is similar in structure to the FPLR rule we described earlier. In fact, the last two lines of Eq. (2.7.1) are identical to the modified SBC rule with fixed points $\{F({\theta }_{D}\le Ca(t)\le {\theta }_{P}) = 0, F(Ca(t)>{\theta }_{P}) =1)\}$ and learning rates $\{\eta ({\theta }_{D}\le Ca(t)\le {\theta }_{P}) = {\eta }_{D}, \eta (Ca(t)>{\theta }_{P}) ={\eta }_{P})\}$. The first two lines of Eq. (2.7.1), however, add a new feature, namely the dependence of the fixed points in the pre-depressive region on the present weight $w$, in addition to the [Ca^2+^].

### Two-dimensional FPLR rule

It is possible to generalize the simplified GB rule into a fully generic two-dimensional FPLR plasticity rule that specifies the fixed points and learning rates as a function of both the synaptic [Ca^2+^] and the current weight. Similar to the SBC rule, we have:

2.8.1 $$\Delta w=\eta \left(Ca\left(t\right),w\right)*\left(F\left(Ca\left(t\right),w\right)-w\right)$$

Here, both the learning rates $\eta$ and the fixed points $F$ are two-dimensional step functions of both the [Ca^2+^] and the current weight $w$, as opposed to a one-dimensional step function of only the [Ca^2+^] in the SBC rule. Usually, we will keep the standard fixed points and learning rates for depression and potentiation as only dependent on the [Ca^2+^], so we still have $F\left({\theta }_{D}<Ca\left(t\right)< {\theta }_{P} \right)=0$ and $F\left(Ca\left(t\right)> {\theta }_{P} \right)=1$. However, for the pre-depressive region, we can now specify an arbitrary number of *weight-dependent* fixed points and their associated learning rates, which allows the synapse to drift to one of several final stable states.

Each fixed point for the weights in the pre-depressive region of calcium has a basin of attraction, i.e. a range of weights surrounding that fixed point that eventually converge to the stable weight. When specifying fixed points of the weights as a function of the present weights, care must be taken to avoid overlapping basins of attraction. For example, if the fixed point for a synapse with a weight of $w= 0.8$ is $w= 1$, the fixed point for weight $w = 0.9$ must also be $w= 1$, because $w= 0.8$ must pass $w= 0.9$ on its way to $w= 1$.

**Fig. 4:**
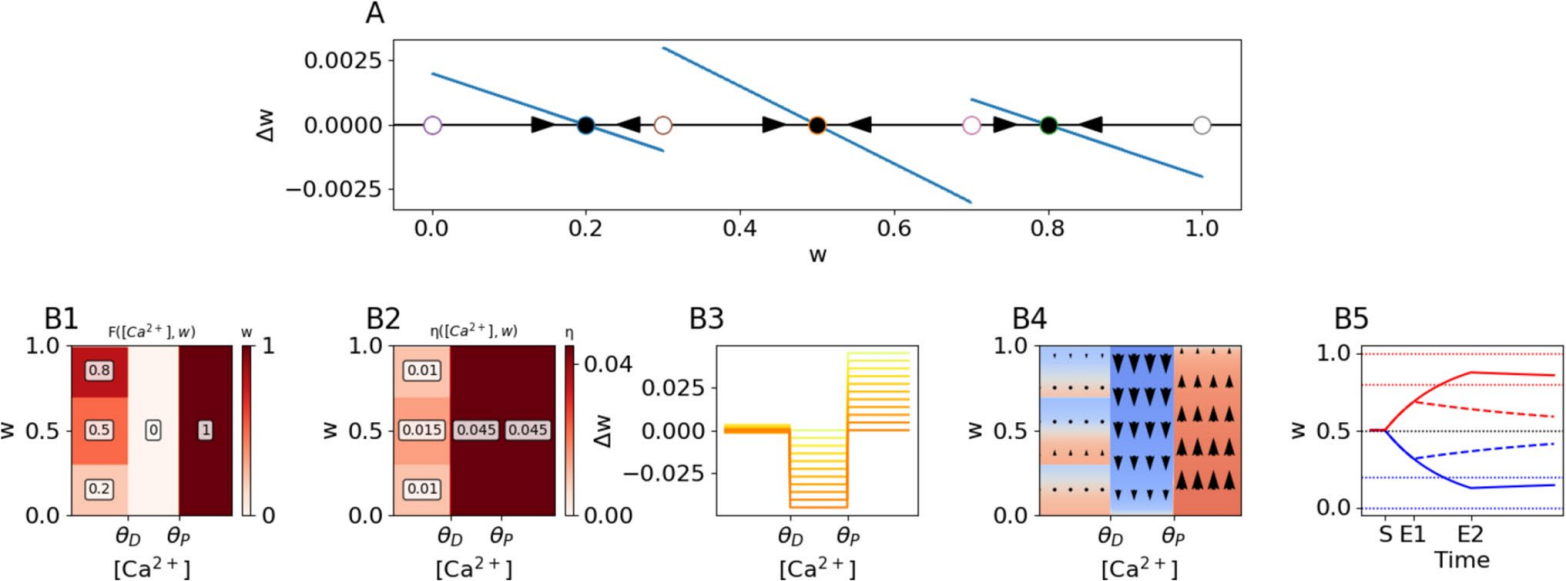
The two-dimensional FPLR rule. **(A)** Defining fixed points and basins of attraction for pre-depressive drift in the two-dimensional FPLR rule. We can define an arbitrary number of stable fixed points (filled circles) as a function of the current weight $w$, as well as the boundaries of their respective non-overlapping basins of attraction (open circles). Blue lines indicate change in weight $(\Delta w)$ as a function of the present weight $w$. **(B1)** Representation of fixed points as a function of both $\left[{Ca}^{2+}\right]$ and current value of the synaptic weight $w$. Values in the pre-depressive region as in (A) **(B2)** Learning rates in two-dimensional FPLR rule as a function of both $\left[{Ca}^{2+}\right]$ and current value of the synaptic weight $w$, note that the learning rate for the MIDDLE fixed point in the pre-depressive region is larger than the rates in the other two regions. **(B3-B4)** Weight dependence and phase plane for rule shown in (B1-B2). **(B5)** A step of calcium of potentiatve (weight response indicated by red trace) or depressive (weight response indicated by blue trace) $\left[{Ca}^{2+}\right]$ is applied for either a short (2 s, dash-dot line, stimulation starts at S and ends at E1), or long (20 s, solid line, stimulation starts at S and ends at E2) duration. The long-duration stimulus escapes the middle fixed point’s basin of attraction; the short duration stimulus does not. Note that the long-duration stimulus overshoots/undershoots the late-phase fixed points

To enforce this constraint, one must assign $$N$$ fixed points and $$N+1$$ boundaries of the basins of attraction within each region of [Ca^2+^] such that the fixed points are always inside the closest basin boundaries on either side (Fig. 4C). For example, we can consider a rule that incorporates a tri-stable pre-depressive drift, where strong synapses drift to an UP state of $$w= 0.8$$, weak synapses drift to a DOWN state of $$w= 0.2$$, and synapses which aren’t particularly strong or weak drift toward a MIDDLE state of $$w= 0.5$$ (instead of an unstable fixed point of $$w= 0.5$$). We intentionally choose fixed points in the pre-depressive region of [Ca^2+^] that are different from those in the potentiative ($$w= 1)$$ and depressive ($$w= 0)$$ regions of [Ca^2+^] to reflect experimental results that synaptic weights during the early phase of LTP and LTD can overshoot/undershoot the eventual weights to which they are stabilized (Manahan-Vaughan et al., 2000; Redondo et al., 2010).

We can specify the fixed points for the pre-depressive calcium region $F\left(Ca\left(t\right)< {\theta }_{D} ,w\right)$ by choosing the fixed points as $\left[0.2, 0.5, 0.8\right]$ and the boundaries of the basins of attraction as $\left[0, 0.3, 0.7,1\right]$ (Because 0 and 1 are the fixed points of early-phase depression and potentiation, the weight will never go above or below those values unless initialized there.) This translates into

2.8.2 $$F\left(Ca\left(t\right)<\theta_D,w\right)=\left\{\begin{array}{lll}0.2,&&0<w<0.3\\0.5,&&0.3<w<0.7\\0.8,&&0.7<w<1\end{array}\right.$$

The boundaries of the basins of attraction can be set as unstable fixed points, so we have that e.g. $F\left(Ca\left(t\right)< {\theta }_{D} ,w=0.3\right)=0.3$. (Half stable fixed points can also be used by employing non-strict inequalities in Eg. 4.3). By specifying the fixed points of the pre-depressive drift in this manner, in the absence of a plasticity stimulus, the synaptic weight will be drawn to one of the three stable fixed points, depending on which of the three basins of attraction it is currently in (Fig. 4A). The fixed points and associated learning rates for the two-dimensional FPLR rule can be visualized as a two-dimensional heatmap (Fig. 4B1, 4B2). We note that the learning rates can vary as a function of the synaptic weight just like the fixed points, so we can set the drift rate toward the MIDDLE fixed point to be faster than the drift rate toward the other fixed points. The weight dependence and phase plane for this plasticity rule are illustrated in Fig. (4B3-4B4).

We can illustrate the dynamics of the tri-stable FPLR rule by applying our canonical protocol of a step of potentiative or depressive calcium for either a short or long duration. A synapse exposed to the short-duration stimulus will briefly potentiate or depress, but insufficiently to escape the MIDDLE fixed point’s basin of attraction, so it drifts back to the MIDDLE position. A synapse exposed to a long-duration stimulus, however, will escape the MIDDLE fixed point’s basin of attraction and thus drift to either the UP or DOWN fixed point after the calcium step is turned off (Fig. 4B5).

To summarize, we have proposed two versions of the FPLR rule: a “one-dimensional” version (Eq. (2.4.1)), based on the work of (Shouval et al., 2002), where the fixed points and learning rates of the synaptic weight dynamics depend only on the calcium concentration, and a “two-dimensional” version, based on the model of (Graupner & Brunel, 2012) (Eq. (2.8.1)), where the fixed points and learning rates depend on the synaptic weight as well as the calcium concentration. We will now elaborate on the biological relevance of the one-dimensional vs. two-dimensional FPLR rules.

### Incorporating late LTP/LTD

One of the major differences between the one-dimensional FPLR rule and the two-dimensional rule pertains to what happens in the “late phase” of LTP/LTD, hours after an LTP/LTD stimulation protocol is completed and [Ca^2+^] levels have returned to baseline. In the one-dimensional rule the weight eventually drifts to 0 (or some other baseline state in the modified SBC rule), while in the two-dimensional rule weights are slowly stabilized to one of several stable states, depending on the synaptic weight at the completion of the plasticity protocol.

Biologically, the one-dimensional rule is more representative of what has been observed in the absence of protein synthesis, and two-dimensional rule can be seen to correspond to the situations where proteins are synthesized to stabilize synaptic strengths for a longer period. In hippocampal cells, late-phase stabilization of potentiated and depressed synaptic states requires synthesis of proteins, without which synapses eventually drift back to their original strengths (Frey & Morris, 1997; Kauderer & Kandel, 2000; Redondo et al., 2010; Sajikumar et al., 2005). A similar phenomenon has been observed for LTD in Purkinje cells (Linden, 1996). This dependence on protein synthesis can be incorporated into the generic GB model by adding another dimension to the step functions for the weights and fixed points.

2.9.1 $$\Delta w=\eta \left(Ca\left(t\right),w\left(t\right), protein\left(t\right)\right)*\left(F\left(Ca\left(t\right),w\left(t\right),protein\left(t\right)\right)-w\left(t\right)\right)$$

For illustration, if we make a simplifying assumption that there is a single stabilizing protein which can be either present (1) or absent (0), we have the following rule:

2.9.2 $$\Delta w\left(Ca(t),w(t),\text{protein}(t)\right) = \left\{ \begin{array}{ll} \eta\left(Ca(t)\right)\left(F\left(Ca(t)\right) - w(t)\right), & \text{protein}(t) = 0 \\ \eta\left(Ca(t),w(t)\right)\left(F\left(Ca(t),w(t)\right) - w(t)\right), & \text{protein}(t) = 1 \end{array} \right.$$

**Fig. 5:**
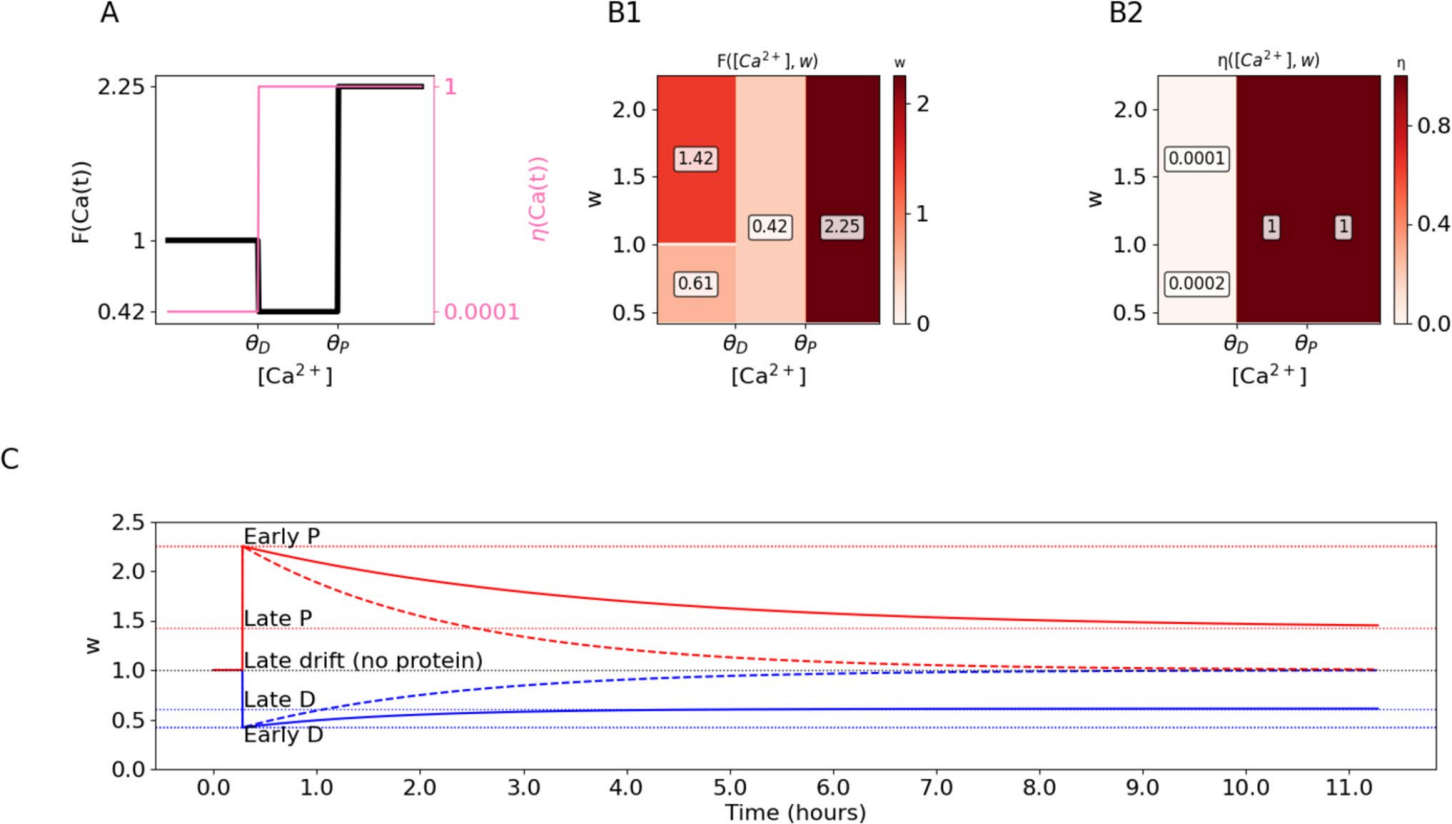
Protein-dependent late phase plasticity.** (A) **In the absence of protein synthesis, fixed points and learning rates (in units of seconds) can be described by one-dimensional functions of the $\left[{Ca}^{2+}\right]$, as in the one-dimensional FPLR rule (Fig. 1F-1G). Here we use biologically realistic values for the late phase of plasticity (described in **Methods**). **(B)** If there are proteins to stabilize the new synaptic weights, fixed points (**B1**) and learning rates (**B2**) can be described as two-dimensional functions of $\left[{Ca}^{2+}\right]$ and the current weight $w$, as in the two-dimensional FPLR rule (Eq. 2.8.1). **(C)** Weight drift over the late phase of plasticity in the presence (solid line) and absence (dashed line) of protein synthesis subsequent to maximally potentiative (red) or depressive (blue) $\left[{Ca}^{2+}\right]$ stimulation in the early phase. Dotted lines indicate fixed points for early/late phase for potentiative (P)/depressive (**D**) stimuli in the presence/absence of protein synthesis. Fixed points and late phase dynamics use the biologically realistic parameters from (A) and (B)

In other words, if there are no proteins to stabilize the new synaptic weights after a plasticity-inducing calcium stimulus, then synaptic weights simply drift to baseline, and the plasticity dynamics in each region of $\left[{Ca}^{2+}\right]$, including the pre-depressive region, can thus be described using a single weight-independent fixed point and learning rate in each region of $\left[{Ca}^{2+}\right]$, as in the generic one-dimensional rule. However, if there are proteins to stabilize the new synaptic weights after a plasticity-inducing calcium stimulus, then fixed points and learning rates in the pre-depressive region of $\left[{Ca}^{2+}\right]$ are weight-dependent and the plasticity dynamics must be described with two-dimensional step functions, as in the two-dimensional rule. We illustrate these protein-dependent late phase dynamics using biologically realistic fixed points and time constants in Fig. 5 (see **Methods**). Because the late phase dynamics operate on the order of hours and the early phase dynamics operate on the order of milliseconds, we can assume for simplicity that the early phase stimulation causes an instantaneous jump to the depressive or potentiative fixed point, i.e. we can set $\eta =1 second$ for the potentiative and depressive regions, while the late phase drift or stabilization occurs 4 orders of magnitude more slowly.

### Modeling frequency and spike timing dependent plasticity

Although the FPLR framework makes the dynamics of calcium-based plasticity more straightforward, it is still quite similar to the original SBC and GB rules, and one would not expect to observe substantial discrepancies in the implementation of plasticity inducing protocols. Nevertheless, for the sake of completeness, we reproduce two classic protocols: frequency-dependent plasticity and spike timing dependent plasticity (STDP). Most of the literature about these protocols pertains to the early phase of plasticity, and we therefore use the version of the one-dimensional FPLR rule (Eq. 2.4.1) where the pre-depressive drift is turned off by setting $\eta \left(Ca< {\theta }_{D}\right)=0$, as in Fig. 1I.

To model the calcium itself, we use the simplified formalism (Graupner & Brunel, 2012) where calcium concentration at a particular spine is modeled as a value which jumps by ${C}_{pre}$ whenever there is a presynaptic spike at that spine, jumps by ${C}_{post}$ whenever there is a postsynaptic spike, and decays toward 0 at the rate of ${\tau }_{Ca}$ (Graupner & Brunel, 2012). Formally, this can be expressed by the equation:

2.10.1 $$\Delta Ca\left(t\right)=Ca\left(t+1\right)- Ca\left(t\right)=-\frac{1}{{\tau }_{Ca}}Ca\left(t\right)+{C}_{pre}{\sum }_{\left\{{t}_{i}\right\}}\delta \left(t-{t}_{i}\right)+{C}_{post}{\sum }_{\left\{{t}_{j}\right\}}\delta \left(t-{t}_{j}\right)$$

where $\left\{{t}_{i}\right\}$ are the times of the presynaptic input spikes at that spine and $\left\{{t}_{j}\right\}$ are the times of the post synaptic spikes.

**Fig. 6:**
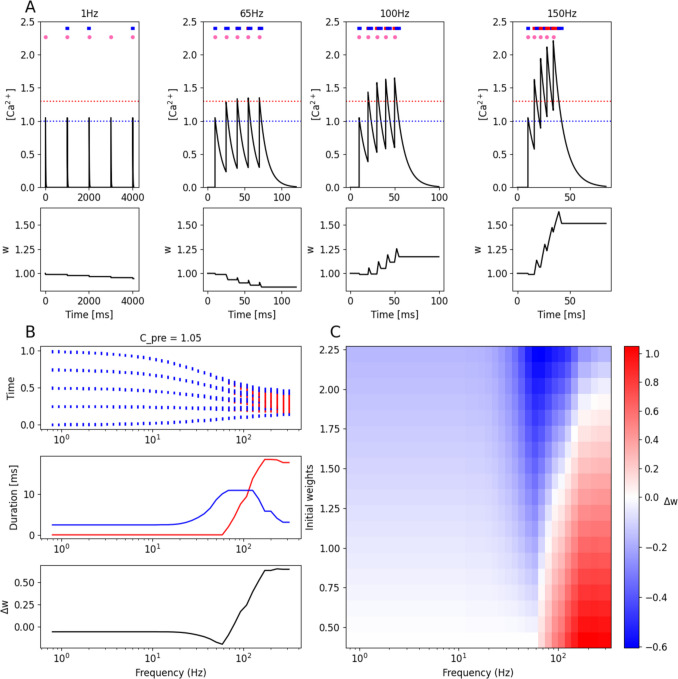
Frequency-dependent plasticity. **(A)** Calcium (top) and weight changes (bottom) produced by a train of 5 presynaptic inputs (pink dots) at different frequencies. Dashed blue and red lines indicate ${\theta }_{D}$ and ${\theta }_{P}$, respectively. Blue and red stripes at the top of the plots form a bar code indicating times at which the calcium is in the depressive (blue) or potentiative regions (red). **(B)** (Top) Bar codes (as in A top) oriented vertically as a function of input frequency. Time axis is normalized to beginning and end of each simulation. (Middle) Total duration in depressive (blue) or potentiative (red) regions of $\left[{Ca}^{2+}\right]$ as a function of input frequency. (Bottom) Total change in weight (final-initial) for each input frequency. **(C)** Heatmap of plastic changes as a function of both initial weight and input frequency

We first demonstrate how calcium-based plasticity results in synaptic changes that depend on the frequency of the presynaptic input. Experimentally, low-frequency stimulation (LFS) tends to produce depression, while high-frequency stimulation (HFS) tends to produce potentiation (O’Connor et al., 2005b). Intuitively, if ${C}_{pre}$ is below ${\theta }_{D}$, a single presynaptic input spike will not produce depression, but if several input spikes occur such that each spike rides on the tail of the previous spike, the $\left[{Ca}^{2+}\right]$ can build up such that it rises above ${\theta }_{D}$, causing depression, and if the spikes occur with sufficiently high frequency, the $\left[{Ca}^{2+}\right]$ can rise above ${\theta }_{P}$, resulting in potentiation (Fig. 6A). To visualize the durations that the $\left[{Ca}^{2+}\right]$ is in each region, it can be helpful to create a “bar code” for different stimulation protocols by indicating the times at which the $\left[{Ca}^{2+}\right]$ was above the ${\theta }_{D}$ and ${\theta }_{P}$, which can help give a more intuitive feel for which protocols will result in synaptic potentiation or depression (Fig. 6A-6B).

Because the synaptic changes in the FPLR framework occur in an asymptotic manner, it is easier to potentiate weak synapses and easier to depress strong synapses. This means that the result of a frequency-dependent plasticity protocol will depend on the initial synaptic weight. If a synapse starts out closer to the depressive fixed point, it is easier to potentiate, while if it starts out closer to the potentiative fixed point, it is easier to depress. In fact, synapses which start out with large weights may even be depressed by a high-frequency protocol (Fig. 6C).

The reason that high-frequency protocols depress strong synapses in this model is subtle: whenever the $\left[{Ca}^{2+}\right]$ rises past ${\theta }_{P}$, it will inevitably spend some time in the depressive region when the stimulation ends and the calcium decays to the baseline (“what goes up must come down”). Thus, for synaptic potentiation to be maintained, the magnitude of potentiation must be sufficiently large to not be completely erased by the subsequent depression. However, if the synapse starts out with a weight near the potentiation fixed point, virtually no potentiation can occur, so the depression during the decay phase of the calcium will be the only effect observed.

The “what goes up must come down” effect is an inevitable quirk of calcium threshold models which incorporate decaying calcium signals, and while this quirk can sometimes be helpful in explaining some experimental results, it creates complications for reproducing other experimental results, and it is also a bit counterintuitive. There is some experimental evidence that potentiation protocols will “lock in” potentiation to prevent subsequent depression (O’Connor et al., 2005b), which may help to alleviate this “what goes up must come down” problem. Our simulations here, however, do not include a lock-in feature, so potentiation protocols will always include a period of depression once the stimulation concludes and the $\left[{Ca}^{2+}\right]$ decays back to baseline. It is also possible to avoid this issue if the rate of depression is substantially slower than the rate of potentiation and the decay of the calcium is sufficiently fast such that the amount of depression that occurs while the calcium is decaying back to baseline after a potentiation protocol is negligible.

We note that if the postsynaptic neuron is depolarized during the frequency-dependent plasticity protocols, this could increase the calcium influx into the neuron during the presynaptic stimulation via VGCCs and NDMA channels, potentially increasing the duration at which the $\left[{Ca}^{2+}\right]$ is above the plasticity thresholds, and thereby increasing the magnitude of plasticity or reducing the frequency of the stimulation needed to achieve a particular direction or magnitude of plasticity, see (Ngezahayo et al., 2000).

### Spike timing-dependent plasticity (STDP)

We can use our intuition from the above section about frequency-dependent plasticity to build an FPLR-based simulation of spike-timing dependent plasticity (STDP). We wish to replicate the “classic” STDP curve, where presynaptic input before postsynaptic stimulation causes potentiation at the activated presynaptic synapse, whereas postsynaptic spiking before presynaptic input causes depression, and both effects decrease with increased time intervals (Bi & Poo, 1998).

**Fig. 7:**
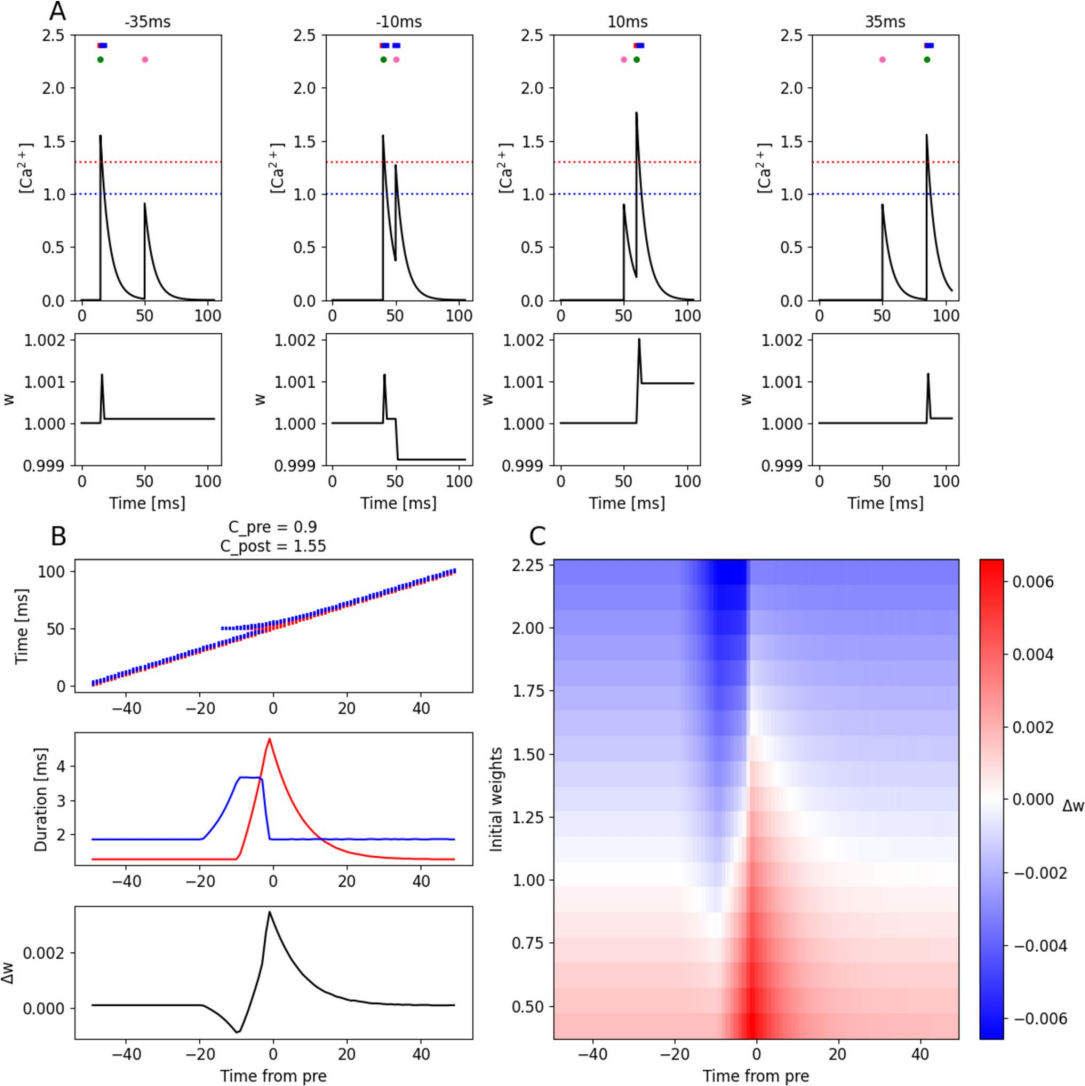
Spike Timing Dependent Plasticity (STDP). **(A)** Calcium (Top) and weight changes (bottom) produced by a single presynaptic (pink dot) and postsynaptic (green dot) stimulation at different time intervals. Negative time interval indicates post-before-pre, positive time interval indicates pre-before-post. Dashed blue and red lines indicate ${\theta }_{D}$ and ${\theta }_{P}$, respectively. Blue and red stripes at the top of the plots form a bar code indicating times at which the calcium is in the depressive (blue) or potentiative regions (red). **(B)** Bar codes (top), duration spent in each $\left[{Ca}^{2+}\right]$ region (middle; blue indicates depressive region, red indicates potentiative region), and weight changes (bottom) as a function of post–pre interval. **(C)** STDP heatmap as a function of post–pre interval and initial weights

To reproduce this result, we simulated the calcium generated by single presynaptic spike (which creates a $\left[{Ca}^{2+}\right]$ pulse of height ${C}_{pre}$) at 50 ms of a 100 ms simulation. We then generate a postsynaptic spike (which creates a $\left[{Ca}^{2+}\right]$ pulse of height ${C}_{post}$) either before or after the presynaptic spike at varying timing intervals (Fig. 7A). By appropriately setting the simulation parameters, it is possible to replicate the classic STDP curve (Fig. 7B). As with the frequency-dependent protocol above, the magnitude of synaptic weight change in the STDP protocol will depend on the initial synaptic weight; large weights are faster to depress and small weights are faster to potentiate (Fig. 7C).

We note that different parameter settings for ${C}_{pre}$*,* ${C}_{post}$, ${\eta }_{P}$, and ${\eta }_{D}$ can produce a variety of STDP curves, as observed in some experiments and discussed in (Graupner & Brunel, 2012). The 'what goes up must come down' phenomenon plays a crucial role in shaping STDP curves. For instance, to achieve the classical STDP curve shown in Fig. 7B-7C, the parameters must be carefully tuned such that for a postsynaptic spike, the magnitude of potentiation induced when the $\left[{Ca}^{2+}\right]$ rises into the potentiative region of $\left[{Ca}^{2+}\right]$ is almost exactly balanced by the magnitude of depression that occurs as the $\left[{Ca}^{2+}\right]$ falls back through the depressive region of $\left[{Ca}^{2+}\right]$; otherwise, a lone postsynaptic spike could cause potentiation or depression.

We note that in many standard STDP protocols, it is required to repeat the pre-post pairings numerous times in order to see a substantial plastic effect (e.g. 60 pairings in the original work from (Bi & Poo, 1998). This is consistent with our model if one assumes that each pre-post pairing stimulus indeed individually induces some small change in weight, but in order for the change in synaptic efficacy to be noticeable (e.g. in order to overcome various other sources of noise in the observed synaptic efficacy), many repetitions of the stimulation are needed. To replicate this effect, we have set the values of ${\upeta }_{\text{P}}$ and ${\upeta }_{\text{D}}$ to be very small relative to the values of the fixed points of the weights (see **Methods**).

### Modeling behavioral time scale plasticity

Recent experimental findings in the hippocampus have revealed a novel form of plasticity, known as behavioral time scale plasticity (BTSP) (Bittner et al., 2015, 2017; Milstein et al., 2021). A mouse running on a treadmill can spontaneously form place cells in the hippocampal CA1 when the soma of a CA1 neuron is injected with a strong current, inducing a plateau-like voltage depolarization in the neuron. After a single induction, this “plateau potential” results in the neuron exhibiting a place field selective to the mouse’s location few seconds before or after the time of the plateau potential. Moreover, this place field can be modified; if a second plateau is induced while the mouse is at a different location near the first place field, the place field will shift to the new location, thus “overwriting” the first place field. However, if the second location is sufficiently far away from the neuron’s first place field, the neuron forms an additional place field at the second location without “overwriting” the first place field (Milstein et al., 2021). Although this form of plasticity has been replicated many times, the biological mechanisms underlying it remain unclear. Some extant models propose an “eligibility trace”, i.e. chemical signal that serves to serve as a seconds-long coincidence detector between synaptic input and plateau induction (Cone & Shouval, 2021; Gerstner et al., 2018; Milstein et al., 2021).

One reason why the calcium control hypothesis was not considered as a mechanism for BTSP in previous work is that classical calcium-based protocols like STDP occur at timescales that are faster than would be necessary to model BTSP (Cone & Shouval, 2021; Gerstner et al., 2018; Milstein et al., 2021). Moreover, recent work has shown that calcium indicators with low binding affinity show fast decay constants, on the order of tens of milliseconds (Miyazaki & Ross, 2022). Indeed, in our own replications of STDP and frequency-dependent plasticity, we used time constants for the decay of calcium on the order of 10 ms, while modeling BTSP required calcium decay time constants on the order of seconds.

Despite these timescale differences, however, given the extensive evidence for the calcium basis of plasticity in other contexts, and the fact that NMDA and VGCC channel blockers disrupt BTSP (Bittner et al., 2017; Caya-Bissonnette et al., 2023), it is still reasonable to posit that calcium is the key ingredient underlying coincidence detection in BTSP. We therefore suggest that intracellular calcium-induced calcium release (CICR) from internal calcium stores in the endoplasmic reticulum (ER) (Benedetti et al., 2024; Friel & Tsien, 1992; Rose & Konnerth, 2001), can titrate the speed at which calcium decays, enabling calcium-based plasticity to operate on a spectrum of timescales.

**Fig. 8:**
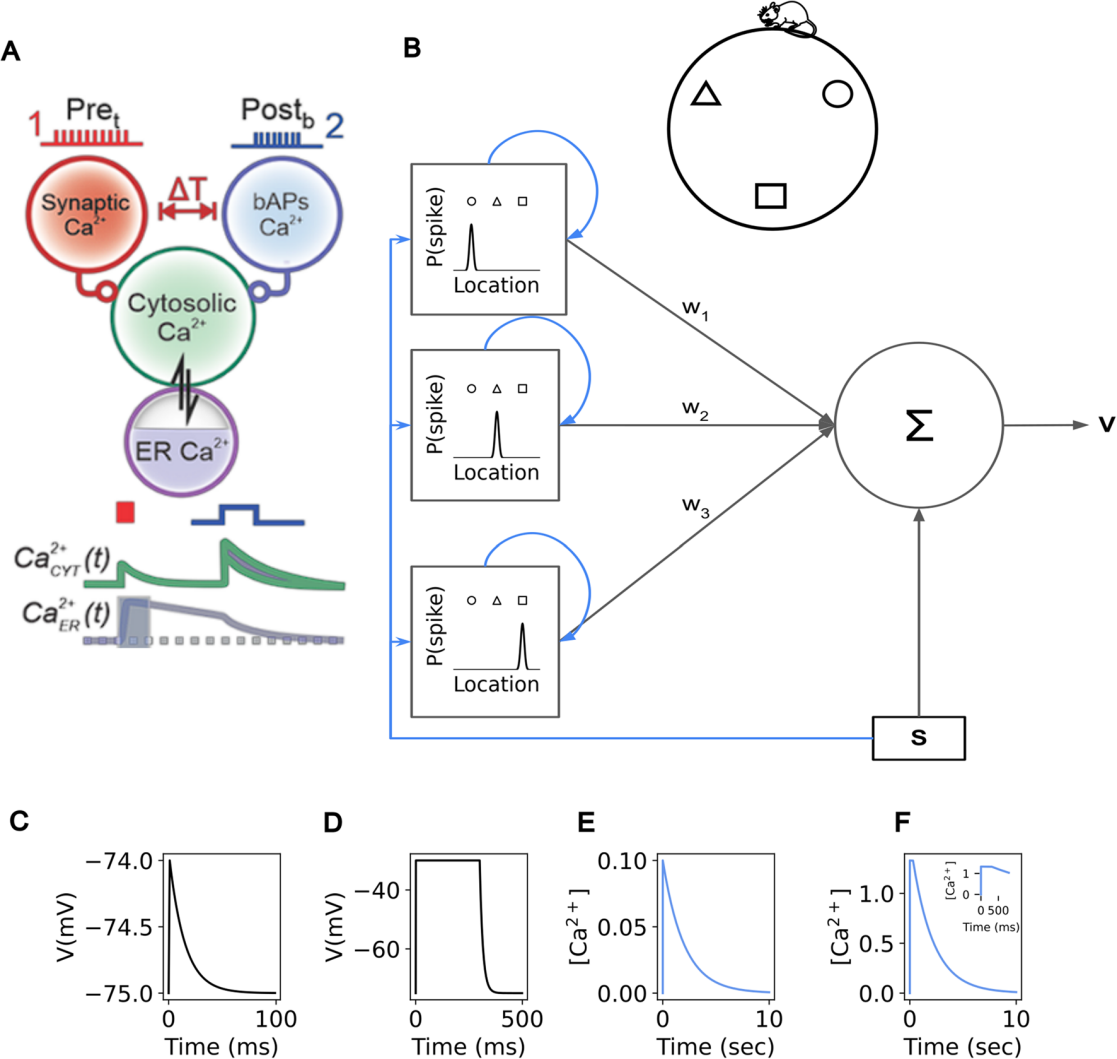
Simulation and neuron model for behavioral time-scale plasticity.** (A)** Schematic of integration of calcium signals over long timescales via the endoplasmic reticulum (ER), adapted from (Caya-Bissonnette et al., 2023). When a $\left[{Ca}^{2+}\right]$ influx-inducing event occurs (i.e. presynaptic stimulation or a postsynaptic burst), $\left[{Ca}^{2+}\right]$ enters the cytoplasm (${\left[{Ca}^{2+}\right]}_{CYT}$, green) as well as internal storage in the ER (${\left[{Ca}^{2+}\right]}_{ER}$, blue trace). Although the cytoplasmic $\left[{Ca}^{2+}\right]$ decays quickly, the decay time constant for the ${\left[{Ca}^{2+}\right]}_{ER}$ is substantially longer. When the second $\left[{Ca}^{2+}\right]$ influx-inducing event occurs, calcium is released from the ER via calcium-induced calcium release (CICR), summing with the cytosolic calcium. **(B)** (Top) Schematic of a mouse running along a circular track. Shapes indicate position along the track. (Bottom) A leaky integrator neuron sums weighted current input (black arrows) from each of its spatially-tuned synapses (squares). Spatial input also induces calcium (blue arrows) locally at each synapse. A supervising signal (S) can induce a plateau potential in the postsynaptic neuron as well as globally broadcast calcium to all synapses simultaneously. **(C)** Voltage response of the neuron to a single presynaptic spike. **(D)** Voltage response of the neuron to a supervisor-induced plateau potential. **(E)** Calcium response of a synapse to a single presynaptic spike at that synapse. The long timescale of this decay is a simplification of the ER-based model in (A), where we treat the sum ${\left[{Ca}^{2+}\right]}_{CYT}+{\left[{Ca}^{2+}\right]}_{ER}$ as a single calcium trace with one long time constant (~ 2s).** (F)** Calcium response at one synapse to a supervisor-induced plateau potential. Inset: zoom-in on the first 800 ms to show calcium plateau. Trace here is a simplification of ${\left[{Ca}^{2+}\right]}_{CYT}+{\left[{Ca}^{2+}\right]}_{ER}$, as in (E)

Indeed, recent experimental and modeling studies have demonstrated the importance of intracellular calcium release for BTSP (Caya-Bissonnette et al., 2023; Jain et al., 2024; O’Hare et al., 2022). In particular, the work of (Caya-Bissonnette et al., 2023) proposes that presynaptic or postsynaptic events (i.e. synaptic stimulation or a postsynaptic burst) can trigger influx of calcium into intracellular calcium stores in the endoplasmic reticulum (ER). The calcium stored in the endoplasmic reticulum may decay at slower timescales than the calcium in the cytosol, on the order of seconds. If the ER was filled within the last several seconds by a calcium influx-inducing event, a subsequent calcium influx-inducing event can stimulate the calcium previously stored in the ER to be released into the cytosol via calcium-induced calcium release (CICR). This ER-sourced calcium can then sum with the influx of extracellular calcium induced by the second event to induce plasticity (Fig. 8A).

As a simplification of this mechanism, we can think of the sum of the cytosolic calcium and the internally-stored calcium in the ER as a single calcium trace dominated by a single decay time constant, namely the longer (on the order of seconds) decay time constant of the ER calcium. Although this simplification will not be sufficient to replicate all aspects of BTSP, we can use it, together with the FPLR rule, to provide insight into the weight-dependent dynamics of BTSP.

The basic intuition for how calcium control can result in BTSP is similar to that of Hebbian plasticity. Neither the presynaptic input nor the plateau potential themselves bring enough calcium into a spine for a long enough time to induce substantial changes to the synaptic weight. When the $\left[{Ca}^{2+}\right]$ traces from both the presynaptic sensory input and plateau potential coincide (or more precisely, when the $\left[{Ca}^{2+}\right]$ stored in the ER consequent to the synaptic input is released into the cytoplasm after a burst occurs, or vice versa), the combined $\left[{Ca}^{2+}\right]$ from both sources rises above the potentiation threshold for around a second, inducing potentiation at the synapses that were active at the time of plateau induction. Because the potentiated sensory inputs all correspond to the mouse’s location at the time of plateau induction, these potentiated synapses comprise a place field at that location.

The reason why a second induction near to the location of the first induction will overwrite the first place field is because the partially decayed calcium trace from the plateau combines with the calcium from the presynaptic input corresponding to the first place field to surpass the depression threshold but not the potentiation threshold, depressing the synapses from the first place field. (This is if the second place field precedes the first place field on the track. If the first place field precedes the second place field on the track, the depression results from the decaying local calcium signal from the presynaptic input from the first place field combining with the $\left[{Ca}^{2+}\right]$ from the plateau potential.) If the two locations are far enough apart, however, the calcium from the plateau potential will have decayed sufficiently such that it will not surpass the depression threshold for a substantial amount of time when added to the local calcium from the presynaptic input (or vice versa). Finally, the reason why only synapses that were previously potentiated are depressed when “overwritten” in the BTSP protocol is because of the weight dependence of the FPLR rule. At baseline, all synapses that do not take part in a place field have weights near the depressive fixed point, so they cannot be further depressed.

To illustrate this, we simulated a mouse running at constant velocity on a circular track by sequentially presenting track locations as inputs to a leaky integrator model neuron with spatially-tuned presynaptic inputs (Fig. 8B). The neuron could also be stimulated with a voltage step, simulating plateau potential induction. Calcium could enter a synapse as a consequence of its local input or due to the supervising signal, which induces a plateau potential in the neuron and globally broadcasts calcium to all synapses (Fig. 8C-8F). The synaptic calcium decayed with a time constant of ~ 2 seconds (s); as described above, this long time constant represents the time constant of the combined calcium in both the endoplasmic reticulum and the cytosol. The synaptic weights of the model neuron implemented the one-dimensional FPLR calcium-based plasticity rule described in Eq. (2.4.1) with the drift rate set to 0 as in the previous simulations.

**Fig. 9:**
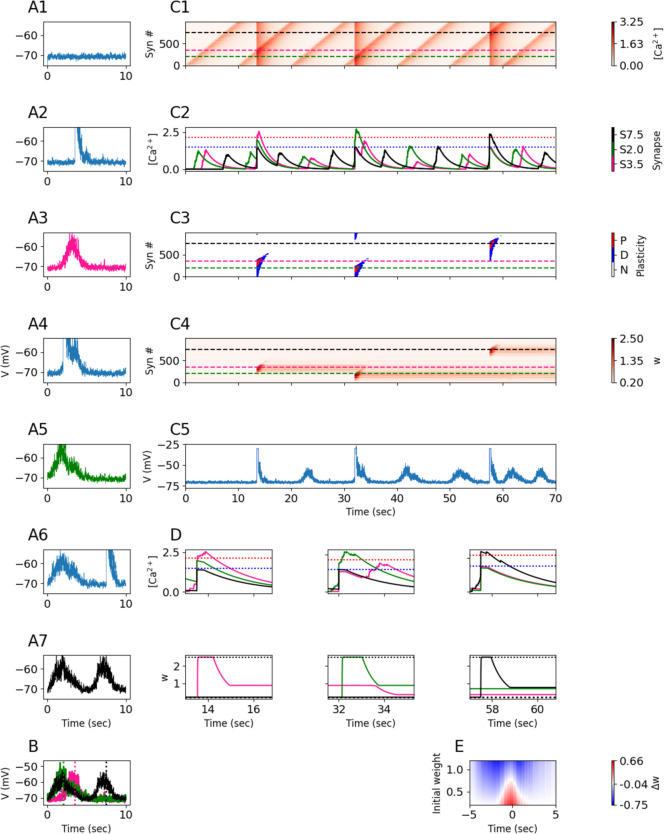
Behavioral time scale plasticity. **(A1-A7)** Voltage traces for 7 laps, each lap lasts for 10 s. A plateau potential is induced in the 2nd, 4th, and 6th laps. Green, pink, and black traces indicate place fields observed on the 3rd, 5th, and 7th laps, respectively. **(B)** Overlay of voltage traces for place fields from the 3rd (pink), 5th (green), and 7th (black) laps. Vertical dotted lines indicate plateau induction location during the preceding lap. **(C1)** Total $\left[{Ca}^{2+}\right]$ per synapse (rows, 40 synapses) over the course of all 7 laps. Dashed lines indicate three synapses whose receptive fields are centered at the location of the first (pink, S3.5, see text) second (green, S2.0) or third (black, S7.5) plateau induction. **(C2)** $\left[{Ca}^{2+}\right]$ traces for the three synapses indicated by the horizontal lines in (C1). Blue and red dotted lines indicate ${\theta }_{D}$ and ${\theta }_{P}$. **(C3)** Plasticity bar codes for each synapse (rows) over all laps. (N: no change, D: depression, P: potentiation) **(C4)** Weights over time for each synapse over all laps. **(C5)** Voltage over time, as in A, for all 7 laps. **(D)** Zoom-in on the calcium traces (top) and weights (bottom) from the three synapses shown in C2 at the time of each plateau induction. In the second lap (left), the pink synapse’s $\left[{Ca}^{2+}\right]$ rises beyond ${\theta }_{P}$, inducing potentiation toward the maximum strength (dotted horizontal line at top), and the green synapse’s $\left[{Ca}^{2+}\right]$ rises above ${\theta }_{D}$, although the green synapse is already at the minimum strength (dotted horizontal line on bottom) so it can’t depress any further. In the fourth lap (middle), the green synapse’s $\left[{Ca}^{2+}\right]$ rises beyond ${\theta }_{P}$, inducing potentiation, and the pink synapses’ $\left[{Ca}^{2+}\right]$ rises above ${\theta }_{D}$, depressing it. In the sixth lap (right), the black synapse’s $\left[{Ca}^{2+}\right]$ rises beyond ${\theta }_{P}$, inducing potentiation. **(E)** Change in weights as a function of initial weight and receptive field distance from plateau onset

The track was presented seven times to the neuron, simulating seven laps, with each lap taking ten seconds to run. At every location on the track, presynaptic inputs whose receptive fields overlapped with that location would contribute current to the neuron as well as induce a calcium signal with height $C_{pre} = 0.1$ at the associated “postsynaptic spine”. The presynaptic calcium signal decayed exponentially at a rate of ${\tau }_{Ca} = 2s$. On the first lap, no induction was performed, establishing a baseline of activity in the absence of a place field. During the second lap, a plateau potential was induced at 3.5 s into the lap depolarizing the neuron’s voltage and inducing a step of calcium for 300 ms with height ${C}_{plateau}= 1.33$ at all synapses. After the plateau induction, the calcium from the plateau also decayed exponentially at a rate of ${\tau }_{Ca}$. During the third lap, a voltage ramp was observed from ~ 1.5–5 s into the lap indicating that the plateau induction from the previous lap had produced a place field. In the fourth lap, a plateau potential was induced at 2 s into the lap. During the fifth lap, a voltage ramp was observed from ~ 0–3.5 s into the lap indicating that the preciously induced place field had been “overwritten” by the plateau induction in the fourth lap. In the sixth lap, a plateau potential was induced at 7.5 s into the lap. During the seventh lap, two voltage ramps were observed, one ramp from ~ 0–3.5 s and the other from ~ 5.5–9 s into the lap indicating that the plateau from the sixth lap induced a new place field but the place field observed at the fifth lap was not erased (Fig. 9A-9B).

To show how the FPLR calcium rule produced these results we visualized the $\left[{Ca}^{2+}\right]$, barcodes and weights of each of the synapses over the entire course of the experiment (Fig. 9C) focusing on the three synapses whose place fields were centered around the track location of the plateau inductions of laps 2, 4 and 6 (Fig. 9C, 9D). We call these synapses S3.5 (pink trace), S2.0 (green trace) and S7.5 (black trace), respectively, according to the track location (in units of seconds from beginning of the mouse’s constant-velocity lap) at the center of their receptive fields. At the time of the first plateau induction, the $\left[{Ca}^{2+}\right]$ for S3.5 surpasses the potentiation threshold for ~ 1 s, potentiating it (and synapses with nearby receptive fields). Because of the “what goes up must come down” effect, S3.5 depresses substantially after it is potentiated due to the $\left[{Ca}^{2+}\right]$ spending some time in the depressive region while it decays. However, S3.5 still remains somewhat potentiated after the calcium fully decays. The $\left[{Ca}^{2+}\right]$ for S2.0 also enters the depressive region, however because all the synapses were initialized at the depressive fixed point, S2.0 cannot be further depressed. This illustrates how the weight dependence of the FPLR rule has important functional consequences for place field formation. Without the weight dependence, there would always be a depressed region around the potentiated location, resulting in a Mexican hat-shaped place field. The weight dependence ensures that only previously-potentiated synapses are ever depressed.

When the second plateau potential is induced in the fourth lap, the $\left[{Ca}^{2+}\right]$ for S2.0 surpasses the potentiation threshold for ~ 1 s, potentiating it (with some subsequent depression) as before. The $\left[{Ca}^{2+}\right]$ of S3.5 enters the depressive region for ~ 1 s due to the combination of the local presynaptic $\left[{Ca}^{2+}\right]$ and the partial $\left[{Ca}^{2+}\right]$ from the decayed plateau potential. This results in the depression of S3.5 back to the depressive fixed point. The strength of S7.5 does not change, as before.

Finally, at the time of the third plateau induction, the $\left[{Ca}^{2+}\right]$ at S7.5 surpasses the potentiation threshold for ~ 1 s, potentiating it. Because the plateau induction at 7.5 s into the track was sufficiently temporally distanced from the activation of the receptive fields of S2.0 and S3.5, the decayed local and supervisory calcium signals did not overlap to surpass the depression threshold for a significant amount of time, resulting in only a negligible magnitude of depression for S2.0 (Fig. 9D).

To ensure that all the above results hold when the locations of the inductions are reversed, we performed a “mirror image” experiment, where plateau inductions at laps 2, 4. and 6 occurred at 6.5 s, 8 s, and 2.5 s from the beginning of the track, respectively. The mirror image experiment yielded similar results (Fig. S1).

To fully characterize the expected weight change induced by a plateau potential for synapses with different initial weights and receptive fields, we simulated a mouse running a single lap with a plateau potential induced in the middle of the lap (at 5 s). Using the $\left[{Ca}^{2+}\right]$ at each synapse we calculated the magnitude and direction of plasticity at each synapse for a range of initial weights. Synapses with receptive fields selective to locations near the center of the track potentiate if they started out with low weights but depress if initialized with large weights (due to the fact that synapses with large weights can’t potentiate much, but do depress when the calcium signal decays because of the “what goes up must come down” effect). Synapses with receptive fields far from the center of the track will depress slightly if they are initialized with large weights but will not appreciably change if initialized with low weights (Fig. 9E). This profile is qualitatively consistent with the experimental results from (Milstein et al., 2021).

## Discussion

In this work, we have developed a straightforward mathematical framework, the FPLR rule, to describe calcium-dependent long-term plasticity dynamics. The FPLR framework, based on the rules of (Graupner & Brunel, 2012; Shouval et al., 2002), enable modelers to describe plasticity dynamics in each region of $\left[{Ca}^{2+}\right]$ by specifying fixed points and learning rates as one-dimensional step functions of the $\left[{Ca}^{2+}\right]$ or as two-dimensional functions of both the $\left[{Ca}^{2+}\right]$ and the current value of the weight. This makes it simple to model novel experimental results such as the examples of Purkinje neurons and additional no-plasticity zones that we showed above. Additionally, we have shown the one-dimensional and two dimensional can be integrated into a single framework wherein SBC-like decay dynamics occur in the late phase of plasticity in the absence of protein synthesis, whereas GB-like stabilization dynamics occur in the late phase when proteins are available to stabilize weight changes made in the early phase of plasticity. The FPLR framework, which allows for an arbitrary number of fixed points, also fits well with the theoretical and experimental literature suggesting that synaptic weights have on the order of 10 discrete states comprised of nanoclusters of AMPA receptors (Bartol et al., 2015; Liu et al., 2017).

To demonstrate the ability of the FPLR framework to reproduce classic plasticity protocols, we used the FPLR approach to implement frequency-dependent and spike timing dependent plasticity. We introduced the technique of plasticity “bar codes” as a simple way of keeping track of the times when the $\left[{Ca}^{2+}\right]$ was in the potentiative or depressive region. We showed that due to the saturating nature of plasticity, the outcome of frequency-dependent plasticity and STDP (indeed, any plasticity protocol) depends on the initial synaptic weights–it is easier to depress strong weights and potentiate weak weights. Finally, we showed that this weight-dependence of the FPLR framework enables us to explain the weight dependence of behavioral time scale plasticity (BTSP).

### Alternative calcium-based plasticity models

We note that both the SBC and GB rules as well as the FPLR framework assume that synaptic weight changes depend on the magnitude of $\left[{Ca}^{2+}\right]$. However, there are other theories as to how plasticity might depend on $\left[{Ca}^{2+}\right]$. The specific source of calcium – i.e. NMDA receptors, voltage-gated calcium channels or internal calcium stores – may affect long term plasticity due to different second messengers being localized to specific “nanodomains” near the different calcium sources (For a review of alternative models, see (Evans & Blackwell, 2015)).

For simplicity, the FPLR model assumes first-order linear dynamics, which makes our model easy to interpret, fit, and simulate. In biology, of course, the underlying processes in plasticity involve more complicated processes at different timescales, and more complex models can capture some of these details. However, since synaptic weight changes observed experimentally often qualitatively seem to follow relatively straightforward asymptotic dynamics, (Redondo et al., 2010), we believe that a first-order linear model such as ours can be sufficient to capture many important properties of plasticity.

More detailed models have also been developed that explicitly incorporate calcineurin and CamKII activity (Li et al., 2023; Rodrigues et al., 2021) as well as other presynaptic and postsynaptic mechanisms (Ebner et al., 2019). It is also possible to explicitly model protein dynamics for plasticity stabilization, for example via synaptic tag-and-capture model (Clopath et al., 2008; Redondo et al., 2010). While more detailed plasticity rules may enable more specific experimental predictions, over 100 molecules have been implicated in long-term plasticity (Sanes & Lichtman, 1999), making it effectively impossible to have truly comprehensive mechanistic model. Our phenomenological framework can thus be useful for creating models that have relatively few parameters while still capturing essential aspects of calcium-based plasticity. Our fixed point–learning rate approach can also be extended to incorporate other molecular mechanisms, as we suggested with protein synthesis-dependent late-phase plasticity, although some molecular processes may not be well-described in this framework. Additional model complexity may also be needed to model plasticity in physiological concentrations of extracellular calcium (Inglebert & Debanne, 2021; Inglebert et al., 2020).

We also note that the original Graupner & Brunel model (Graupner & Brunel, 2012) incorporates stochasticity, where noise in the system causes weights to fluctuate probabilistically between fixed points. This stochastic approach may better capture certain aspects of biological variability in synaptic plasticity. The FPLR framework could be extended to include such stochasticity while maintaining its core asymptotic behavior, potentially offering a more complete description of synaptic dynamics that combines deterministic trends with biological noise.

### Modeling calcium dynamics

Throughout this work, we have used simple exponentially decaying calcium stimuli to demonstrate the dynamics of our plasticity rules. When modeling neurons in a detailed fashion, it will usually be necessary to explicitly model the calcium influx from various sources, such as voltage-gated calcium channels and NMDA receptors, in order to explore the plastic consequences of presynaptic and postsynaptic activity. While detailed modeling of calcium dynamics is beyond the scope of this paper, work in this direction can be found in the original SBC and GB papers (Graupner & Brunel, 2012; Shouval et al., 2002) as well as elsewhere in the modeling literature (Chindemi et al., 2020).

### Modeling behavioral time scale plasticity

As we noted in **Results**, we modeled BTSP assuming that 1) the endoplasmic reticulum could be a mechanism that allows calcium coincidence detection at timescales on the order of seconds, and 2) that we could simplify our representation of the calcium dynamics by modeling only the net calcium, as opposed to separating out the cytosolic and ER calcium. Regarding the former assumption, further experimental work needs to be done to tease out the exact role of the endoplasmic reticulum in BTSP and more precisely quantifying the uptake, release and decay duration of intracellular calcium stores. Regarding the latter assumption, our simplifying assumption of a single, long calcium decay time constant almost certainly does not maintain perfect fidelity to biology. Specifically, because the cytosolic decay is known to occur on millisecond timescales, even if the *coincidence detection* using the endoplasmic reticulum calcium stores may occur on seconds-long timescales, the *plasticity dynamics* themselves, which occur in the cytoplasm when the calcium is above the plasticity thresholds, still presumably use the much shorter cytoplasmic decay rate. Our purpose here was to show how the FPLR rule applied to a very simple model of the calcium dynamics underlying BTSP could produce BTSP-like plasticity profiles; however for more accurate fidelity to biology, more detailed implementation of the separate dynamics of the cytoplasmic and ER-stored calcium are needed, as in the work of (Caya-Bissonnette et al., 2023).

We also note that our entire modeling approach assumes that calcium ultimately underlies the dynamics for BTSP. The leading alternative to this assumption is that it is not calcium itself, but rather second messengers, such as CaMKII and phosphatases, that are the main players for BTSP coincidence detection (Li et al., 2023; Xiao et al., 2023). Recent work, however, has shown that CaMKII is not activated during the BTSP induction protocol but rather subsequent to it. Suggesting that CamKII itself is not a sufficient mechanism for the coincidence detection underlying BTSP (Jain et al., 2024).

### Future directions

The FPLR framework suggests standardized experimental paradigms to characterize plasticity dynamics. The fixed points can be directly observed by maintaining $\left[{Ca}^{2+}\right]$ at different values and observing the weights until they stabilize—these asymptotic weight values are, by definition, the fixed points for those $\left[{Ca}^{2+}\right]$ levels. After the weights stabilize, the $\left[{Ca}^{2+}\right]$ stimulus should be turned off, allowing the weights to trend to their ‘drift’ fixed points (which may differ depending on the weight observed immediately following the plasticity protocol, see above). The learning rates can then be determined by fitting Eq. (2.4.4) to the time course of the weight changes. The number of required fixed points and thresholds can be determined by systematically varying $\left[{Ca}^{2+}\right]$ levels and identifying qualitatively different stabilization points.

Experimentally, there are several protocols that enable manipulation of the intracellular $\left[{Ca}^{2+}\right]$ to desired levels, which can then be used to create step-like stimuli of varying magnitudes to ascertain the plasticity fixed points. One approach involves performing some procedure known to induce calcium entry into the cell (such as depolarization paired with presynaptic tetanization) and then to use a calcium chelator, such as EGTA or BAPTA, to lower the levels of free intracellular $\left[{Ca}^{2+}\right]$ (Cho et al., 2001; Mulkey & Malenka, 1992). Protocols also exist for light-dependent calcium-uncaging, which enables direct manipulation of calcium levels (S. N. Yang et al., 1999). Manipulating extracellular calcium can also be used to titrate the amount of calcium influx during any calcium conductance activation (Mulkey & Malenka, 1992). Simultaneous imaging of calcium with a presynaptic excitation stimulus, such as glutamate iontophoresis (Cormier et al., 2001), can enable experimenters to tune a presynaptic input protocol to create step-like $\left[{Ca}^{2+}\right]$ stimuli.

Without performing the above plasticity protocols until the synaptic weight stabilizes, it is difficult to disambiguate the fixed points and the plasticity rates. A synapse which is potentiated via a high-frequency stimulation (HFS) protocol can be potentiated to an even higher strength by performing another HFS protocol. Thus, merely looking at the peak EPSP value after a standard LTP/LTD protocol is insufficient to determine the potentiative or depressive fixed point (Enoki et al., 2009).

The FPLR framework inherently resists overfitting due to its simple mathematical form—each region of $\left[{Ca}^{2+}\right]$ requires only two parameters (a fixed point and a learning rate) which can be estimated directly from experimental results. This parsimony limits the model's degrees of freedom while maintaining its ability to capture complex plasticity behaviors. Additional fixed points or thresholds should only be added when there is clear experimental evidence for qualitatively distinct plasticity regimes that cannot be captured by simpler versions of the model.

In addition to enabling experimentalists to model their results, the FPLR framework is sufficiently simple and flexible that theoreticians can incorporate them into neuron models of varying levels of complexity, ranging from simple point neuron models to detailed biophysical models. This can open avenues toward understanding how calcium-based plasticity can lead to learning in the single neuron or at network-level resolution. In a different work, we have shown that the FPLR rule can be used in a simple single-neuron model, the calcitron, to implement a wide variety of learning rules (Moldwin et al., 2025).

Performing the necessary experiments to find fixed points and learning rates among different cell types, species, brain regions and even at different dendritic locations within the same neuron can lead to a deeper understanding of long-term plasticity. Having an easily-interpretable\ calcium-based model for plasticity can help us understand the interplay between dendritic inhibition and plasticity (Bar-Ilan et al., 2012) as well the interaction between homeostatic plasticity and long-term plasticity (Rabinowitch & Segev, 2006, 2008). The flexibility of our framework to specify arbitrary fixed points and plasticity rates can also enable the exploration of activity-dependent changes in plasticity rules, known as metaplasticity (Abraham, 2008).

## Methods

All figures were created in python using the Numpy (Harris et al., 2020) and Matplotlib (Hunter, 2007) packages.

### Plasticity parameter estimates

Everywhere in the paper except for Fig. 5, learning rates and fixed points are meant to convey qualitative understanding of the dynamics of plasticity, not biologically realistic parameters.

For Fig. 5, we used the following experimental results to approximate biologically realistic time constants: in rat hippocampus, for LTD (in vivo), in the early phase of depression, synapses depress to 42% of their initial strength after initializing a low-frequency stimulation (LFS) (900 pulses at 1 HZ) and either drift back to $\sim$ 92% of their baseline in the presence of anisomycin after 3–4.5 h (which blocks protein synthesis) or stabilize at $\sim$ 61% of their baseline in the absence of anisomycin after 3–4.5 h (Manahan-Vaughan et al., 2000). For LTP (in vitro), in the early phase of potentiation, synapses potentiate to $\sim$ 225% of their initial strength minutes after initializing a high-frequency stimulation protocol (100 pulses at 100 Hz, performed 3 times) high-frequency stimulation (HFS) and either drift back to baseline in the presence of anisomycin after 10 h or stabilize at $\sim$ 142% of their baseline in the absence of anisomycin after 10 h (Redondo et al., 2010).

To calculate the time constants, we assumed that the calcium signal from each pulse lasted ~ 10 ms. As such, the calcium signal lasted ~ 3 s and the LTD calcium signal lasted ~ 9 s.

Because different time constants were found for the drift back to baseline for LTP and LTD in the presence of anisomycin, we used the mean of the learning rates that were calculated (0.0046) from both experiments, although in principle it is possible to use a 2-dimensional rule to differentiate the drifts down from the potentiated state or up from the depressed state even in the absence of protein synthesis.

The fixed points for the weights in Fig. 5 were not precisely calibrated to experimental data due to substantially different baselines in peak EPSP measured in various plasticity experiments (Enoki et al., 2009; Manahan-Vaughan et al., 2000; Redondo et al., 2010). The values used for fixed points in Fig. 5 can be thought of as “change relative to baseline” where value of 1 indicates no change. When modeling experimental data, depending on the context, “synaptic weights” may refer to AMPA conductance, number of AMPA receptors/nanoclusters, integral or peak EPSP or EPSC measured at the dendrite or the soma, spine head volume or area, number of docked vesicles, release probability, or other parameters; fixed point values can be expressed directly in units of the relevant parameters.

### Behavioral time scale plasticity simulation

To simulate behavioral time-scale plasticity (BTSP), we used a leaky integrator neuron where the voltage $V$ is determined by the differential equation:

4.2.1 $${C}_{m}\frac{dV}{dt}= {I}_{leak}(t)+{I}_{syn}(t)+{I}_{plateau}(t)$$

where ${C}_{m}=1\text{ nF},$ ${I}_{leak}$ is the leak current, ${I}_{syn}$ is the total contribution to the postsynaptic neuron from all presynaptic inputs, and ${I}_{plateau}$ is the current contribution from a plateau potential induction. (As we are mainly interested in the subthreshold voltage activity rather than postsynaptic spikes, this model neglects postsynaptic spiking activity). The leak current is defined as:

4.2.2 $${I}_{leak}(t)=-{g}_{L}(V\left(t\right)-{V}_{rest})$$

where ${g}_{L}$ is the leak conductance. ${V}_{rest}$ is the resting potential of the membrane. It is helpful to define the voltage leak time constant ${\tau }_{V} =\frac{{C}_{m}}{{g}_{L}}$. In the absence of input, the voltage thus decays according to the equation $\frac{dV}{dt}= -\frac{1}{{\tau }_{V}}\left(V\left(t\right)-{V}_{rest}\right)$

The presynaptic current is defined as:

4.2.3 $${I}_{syn}(t)=\sum_{i=1}^{N}{w}_{i}*{s}_{i}\left(t\right)$$

where ${w}_{i}$ is the weight of synapse $i$ and ${s}_{i}\left(t\right)$ is a binary variable indicating whether synapse $i$ produced a spike at time $t$. The probability of a presynaptic spike at synapse $i$ was defined as:

4.2.4 $${p(s}_{i}=1) \sim Bernoulli({f}_{i}(t))$$

where ${f}_{i}\left(t\right)$ is a bell-shaped receptive field centered around the neuron’s preferred track location ${l}_{i}$. (We can define locations in terms of milliseconds of time from the start of the lap because we assume the mouse runs at constant velocity.) If the mouse’s location is given as $l\left(t\right)=t mod T$, where $T$ is the track length in units of milliseconds of running time, we have:

4.2.5 $${f}_{i}\left(t\right)={P}_{max}*{e}^{-\left(\frac{{\left({l}_{i}. -l\right)}^{2}}{{r}^{2}}\right)}$$

where ${P}_{max}$ is the probability of the neuron firing a spike when the mouse is at the center of the receptive field of that neuron, and $r$ determines receptive field width. The presynaptic receptive fields thus tile the track length with one receptive field center every $\frac{T}{N}$ milliseconds of running distance.

To ensure that the plateau potential took the form of a rectangular voltage clamp step, we defined the plateau current as:

4.2.6 $$I_{plateau}\left(t\right)=(-I_{leak}\left(t\right)-I_{syn}\left(t\right)-V\left(t\right)+V_{plateau})\ast\sum\limits_{{t}_{i}}\delta\left(t-{t_i}\right)$$

where ${V}_{plateau}$ is the target steady state voltage during the plateau induction and ${\{t}_{i}\}$ is the set of all times at which the plateau induction is active.

The $\left[{Ca}^{2+}\right]$ at each synapse $i$*,* ${Ca}^{i}$, is the sum of the local, presynaptically-induced calcium, ${Ca}_{pre}^{i},$ and the global, plateau-induced calcium, ${Ca}_{plateau}$.

4.2.7 $${Ca}^{i}={Ca}_{pre}^{i}(t)+{Ca}_{plateau}(t)$$

The presynaptic calcium is modeled as a pulse of calcium with an initial concentration of $pr{e}_{height}$, which exponentially decays with a time constant of of ${\tau }_{Ca}$:

4.2.8 $$\frac{{dCa}_{pre}}{dt}= -\frac{Ca\left(t\right)}{{\tau }_{Ca}}+pre\_height*{s}_{i}(t)$$

The postsynaptic calcium is modeled as a rectangular step of calcium of height $platea{u}_{height}$ which also decays to baseline at a rate of ${\tau }_{Ca}$ once the induction ends:

$$\frac{{dCa}_{plateau}}{dt}=-\frac{{Ca}_{plateau}\left(t\right)}{\tau_{Ca}}+\left(plateau_{height}+\frac{{Ca}_{plateau}\left(t\right)}{\tau_{Ca}}\right)\ast\sum\limits_{t\_i}\delta\left(t-{t_i}\right)$$

The parameters for BTSP were fitted using the differential evolution algorithm from SciPy (Virtanen et al., 2020) with a cost function designed to qualitatively reproduce the basic experimental results from (Milstein et al., 2021), (i.e. to qualitatively obtain the results shown in Fig. 9).

## Simulation parameters

### Frequency-dependent plasticity

**Table:** 

Parameter	Value
${Ca}_{pre}$	1.05
${w}_{0}$	1
${\tau }_{Ca}$*	10 ms*
${\theta }_{D}$	1
${\theta }_{P}$	1.3
${\eta }_{D}$	0.04
${\eta }_{P}$	0.055
${F}_{D}$	0.42 (Manahan-Vaughan et al., 2000)
${F}_{P}$	2.25 (Redondo et al., 2010)
$dt$(step size for numerical integration)	0.01 ms

### STDP

**Table:** 

Parameter	Value
${Ca}_{pre}$	0.9
${Ca}_{post}$	1.55
${w}_{0}$	1
${\tau }_{Ca}$	7 ms*
${\theta }_{D}$	1
${\theta }_{P}$	1.3
${\eta }_{D}$	0.001
${\eta }_{P}$	0.00075
${F}_{D}$	0.42
${F}_{P}$	2.25
$dt$	0.01 ms

### BTSP

**Table:** 

Parameters	Value
$N$	1000
$r$	400
$V\_rest (mV)$	−75
$V\_plateau (mV)$	−30
$pre\_height$	0.1
$plateau\_height$	1.33
$plateau duration (ms)$	300
${\tau }_{Ca}\left(ms\right)$	2000
${\tau }_{V}\left(ms\right)$	15
${P}_{max}$	0.02 (Huxter et al., 2003)
$T \left(ms\right)$	10,000
${\theta }_{D}$	1.5
${\theta }_{P}$	2.15
${\eta }_{D}$	0.0017
${\eta }_{P}$	0.15
${F}_{D}$	0.2
${F}_{P}$	2.5

## Supplementary Information

Below is the link to the electronic supplementary material.

Supplementary file1 (DOCX 705 KB)

## Data Availability

All code for this paper can be found at https://github.com/tmoldwin/FPLR.

## Code availability

No datasets were generated or analysed during the current study.
