# Supplementary material for: A generalized mathematical framework for the calcium control hypothesis describes weight-dependent synaptic plasticity
Source: J Comput Neurosci. 2025 Mar 18;53(2):333–57. doi: 10.1007/s10827-025-00894-6 (PMC12181224; doi:10.1007/s10827-025-00894-6)
Supplement: Supplementary file 1 — Supplementary file1 (DOCX 705 KB) [file 10827_2025_894_MOESM1_ESM.docx]

# Extended Data Figure 9_1

| 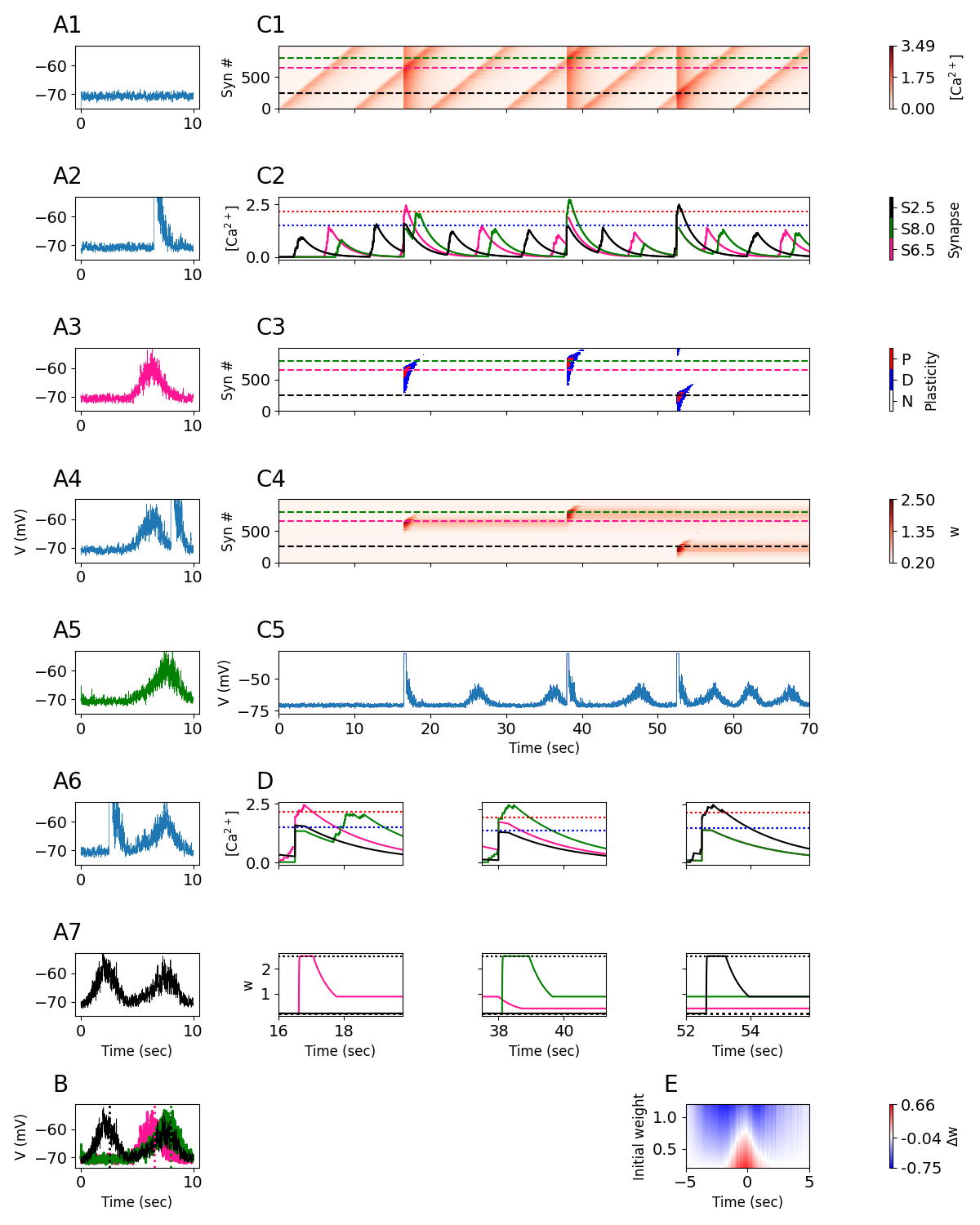 |
| --- |
| **Extended Data Figure 9_1: Behavioral Time Scale Plasticity “Mirror Image” Experiment**  **(A1-A7)** Voltage traces for 7 laps, each lap lasts for 10 seconds. A plateau potential is induced in the 2nd , 4th, and 6th laps. Green, pink, and black traces indicate place fields observed on the 3rd, 5th, and 7th laps, respectively. **(B)** Overlay of voltage traces for place fields from the 3^rd^ (pink), 5^th^ (green), and 7^th^ (black) laps. Vertical dashed lines indicate plateau induction location during the preceding lap. **(C1)** Total $\left[ {Ca}^{2+} \right]$ per synapse (rows, 40 synapses) over the course of all 7 laps. **(C2)** $\left[ {Ca}^{2+} \right]$ over time for three synapses whose receptive fields are centered at the location of the first (pink – S6.5) second (green – S8) or third (black -- S2.5) plateau induction. Boxes show calcium traces around the time of plateau induction (see (D)). **(C3)** Plasticity bar codes for each synapse (rows) over all laps. **(C4)** Weights over time for each synapse over all laps. **(C5)** Voltage over time, as in A, for all 7 laps. **(D)** Zoom-in on the calcium traces (Top) and weights (Bottom) from the three synapses shown in (C) at the time of each plateau induction. In the second lap (left), the pink synapse’s (S6.5) $\left[ {Ca}^{2+} \right]$ rises beyond $\theta_{P}$, inducing potentiation toward the maximum strength (dotted horizontal line at top), and the green synapse’s (S8) $\left[ {Ca}^{2+} \right]$ rises above $\theta_{D}$, although the green synapse is already at the minimum strength (dotted horizontal line on bottom) so it can’t depress any further. In the fourth lap (middle), the green synapse’s $\left[ {Ca}^{2+} \right]$ rises beyond $\theta_{P}$, inducing potentiation, and the pink and black synapses’ $\left[ {Ca}^{2+} \right]$ rises above $\theta_{D}$, depressing it. In the sixth lap (right), the black synapse’s $\left[ {Ca}^{2+} \right]$ rises beyond $\theta_{P}$, inducing potentiation. **(E)** Change in weights as a function of initial weight and receptive field distance from plateau onset. |
